# Influencing factors, gender differences and the decomposition of inequalities in cognitive function in Chinese older adults: a population-based cohort study

**DOI:** 10.1186/s12877-024-04857-x

**Published:** 2024-04-25

**Authors:** Ciran Yang, Zongfu Mao, Shaotang Wu, Shicheng Yin, Yu Sun, Dan Cui

**Affiliations:** 1 School of Public Health, Wuhan University, 115# Donghu Road, 430071 Wuhan, China; 2 Global Health Institute, Wuhan University, Wuhan, China; 3 Dong Fureng Institute of Economic and Social Development, Wuhan University, Wuhan, China

**Keywords:** Chinese older adults, Cognitive function, Recentered influence function (RIF), Inequality, Gender differences

## Abstract

**Background:**

Evidence remains limited and inconsistent for assessing cognitive function in Chinese older adults (CFCOA) and inequalities in cognitive function in Chinese older adults (ICFCOA) and exploring their influencing factors and gender differences. This study aimed to identify influencing factors and inequality in CFCOA to empirically explore the existence and sources of gender differences in such inequality and analyse their heterogeneous effects.

**Methods:**

Based on data from the China Health and Retirement Longitudinal Study (CHARLS) for three periods from 2011 to 2015, recentered influence function unconditional quantile regression (RIF-UQR) and recentered influence function ordinary least squares (RIF-OLS) regression were applied to assess influencing factors of CFCOA, while grouped treatment effect estimation, Oaxaca-Blinder decomposition, and propensity score matching (PSM) methods were conducted to identify gender differences in ICFCOA and influencing factors, respectively.

**Results:**

The results showed heterogeneous effects of gender, age, low BMI, subjective health, smoking, education, social interactions, physical activity, and household registration on CFCOA. Additionally, on average, ICFCOA was about 19.2–36.0% higher among elderly females than among elderly males, mainly due to differences in characteristic effects and coefficient effects of factors such as marital status and education.

**Conclusions:**

Different factors have heterogeneous and gender-differenced effects on CFCOA and ICFCOA, while the formation and exacerbation of ICFCOA were allied to marital status and education. Considering the severe ageing and the increasing incidence of cognitive decline, there is an urgent need for the government and society to adopt a comprehensive approach to practically work for promoting CFCOA and reducing ICFCOA.

**Supplementary Information:**

The online version contains supplementary material available at 10.1186/s12877-024-04857-x.

## Introduction

In a reality where multiple complex factors are constantly intertwined and changing, the promotion of health equity for the whole population is an enduring dimension and a fundamental undertaking in the reform and development of healthcare systems worldwide [1]. Older adults deserve more attention for their health inequalities as a vulnerable group in socioeconomic life because their health vulnerabilities are more evident due to their declining physiological functions and impaired mobility [2]. Moreover, with socioeconomic development and the increase in life expectancy per capita, population ageing is rapidly evolving at an unprecedented rate worldwide and is becoming a serious challenge that all countries cannot ignore [3]. In this context, reducing health inequalities among older adults has become an urgent goal of most modern societies and a barrier to development that they must work to overcome [4].

In fact, health inequalities in older adults are multidimensional, not only in physical function and activity but also in cognitive function. Healthy cognitive function is an important component of healthy and successful ageing for older individuals [5]. The decline of cognitive function in older adults (CFOA) will expose them to more health risks [6], which are directly related to their quality of life and living in later years, and also involve the burden of supporting older adults and medical costs for the whole family and society [7]. China has the largest elderly population in the world, and the population growth curve of the number of older adults will be on the rise for some time to come [8, 9]. Some studies have shown that the prevalence of cognitive impairment among Chinese adults aged 60 years and older exceeds 20% [10], and the burden of disease and economic expenditure associated with cognitive decline in Chinese society is expected to continue to increase in the foreseeable future [11]. Under this situation, the cognitive function of Chinese older adults (CFCOA) and the inequalities in cognitive function of Chinese older adults (ICFCOA) will become increasingly pronounced [12] and important public health issues of particular concern to Chinese society, whose resulting problems will pose significant challenges to society, including families and communities, and will be detrimental to the healthcare system [13, 14].

Based on this, we focus our research on CFCOA and ICFCOA and try to identify specific influencing factors through empirical analysis. We also hope to find out whether there are gender differences in ICFCOA and the possible sources of such gender differences to enrich the whole research work and provide some empirical support to the government and society in promoting the cognitive health and well-being of older adults in the new era.

## Research design

### Data sources

The data used in this study come from the China Health and Retirement Longitudinal Study (CHARLS). CHARLS is a large interdisciplinary survey project conducted by the National School of Development at Peking University. The national baseline survey began in 2011 and included 17,708 respondents from 450 townships or villages in 150 counties in 28 provinces (including autonomous regions and municipalities) using a multistage probability sampling method, with follow-up interviews in 2013, 2015, and 2018. Through scientific and standardised sampling design, large sample size, and rigorous survey implementation, CHARLS has finally developed a high-quality microdata set that is representative of Chinese middle-aged and older adults and their families, and provides highly valuable and realistic information for interdisciplinary research on population ageing in China.

The most recent survey data, i.e., the 2018 data published by CHARLS, did not collect information on the biological nature of respondents, so we only used data from the previous three years for the analysis. According to practical needs, after matching and combining 2011, 2013, and 2015 data, we excluded the sample of respondents who were younger than 60 years old or had missing key variables or no valid responses (including obvious anomalous data and other cases) and the final sample of 4868 observations was obtained in our study.

### Variable measurements

#### Explained variable: CFCOA

CHARLS tests and assesses CFCOA primarily through a live, face-to-face approach with respondents, covering three domains: episodic memory, attention and numeracy, and spatial visualization ability [15]. Episodic memory was measured by reading 10 Chinese nouns at a slow and steady rate and then asking respondents to report the number of Chinese nouns they could successfully recall immediately and after a delay. The mean number of Chinese nouns recalled immediately and after a delay was then used to measure the respondent’s memory level on a scale of 0 to 10 [16, 17]. The attention and arithmetic test consisted of 9 questions that assessed respondents’ knowledge of the time of the survey (including year, month, day, and week information) and their ability to process simple numbers (or the “series 7 test,” in which respondents were asked to answer the result of subtracting 7 from the number 100 and subtracting 7 four times from the remainder of the previous calculation), and the number of questions they were able to answer correctly was used to score this section on a scale of 0 to 9 [18]. The spatial visual ability test is administered by graphic drawing, where the investigator shows the respondent a picture of two overlapping pentagons, and if the respondent can successfully draw a similar figure, a score of 1 is recorded, otherwise a score of 0 is recorded [19].

Following the practice of existing studies [15, 16], the three types of assessment scores were summed to form a comprehensive measure of CFCOA, on a scale of 0 to 20, with higher scores indicating a better CFCOA and vice versa.

#### Explanatory variable

To minimise estimated bias due to omitted variables, relevant variables covering a wide range of individual biological characteristics, lifestyle and socioeconomic status, and family circumstances were included based on existing studies.

The biological factors include gender, age, body mass index (BMI), waist circumference, and subjective health, which are used to provide a comprehensive picture of the respondent’s health and physical condition. BMI was calculated as the ratio of the square of the respondent’s weight to height, while samples with BMI > 60 kg/m^2^ or BMI < 12 kg/m^2^ were considered outliers and excluded [20, 21]. In addition, we converted the continuous variable BMI into categorical variables: light (BMI < 18.5 kg/m^2^), normal (18.5 kg/m^2^ ≤ BMI < 24 kg/m^2^), heavy (24 kg/m^2^ ≤ BMI < 28 kg/m^2^), and obese (28 kg/m^2^ ≤ BMI), according to relevant Chinese guidelines and research practices [22]. We also kept waist circumference as a continuous variable, which was truncated at 1% above and below all samples to avoid the effect of extreme values. Subjective health perceptions were measured by respondents answering the question “How do you feel about your health?”, with results summarised into three categorical variables “poor”, “moderate”, and “excellent”.

Lifestyle factors mainly included respondents’ marital status, smoking, and alcohol consumption. Marital status is a dichotomous variable, with “separated/divorced/widowed/unmarried” being considered “not in marriage”, while “married/cohabiting” are considered “in marriage”. The frequency of smoking was categorised into three variables, including “never,” “former,” and “long-term/current”. The frequency of alcohol consumption was also set as a trichotomous variable, including “never”, “less than once a month”, and “more than once a month”.

Socioeconomic and family factors include educational attainment, social interaction, physical activity, family size, family socioeconomic status, and type of household registration. Educational attainment was categorised as “no formal education,” “elementary school or below,” and “junior high school or above”. Social interaction is a dichotomous variable and is considered “active” and is assigned a value of 1 if the respondent has participated in at least one of the social activities in the past month listed by the investigator, otherwise it is considered “inactive” and assigned a value of 0. Physical activity is recorded as vigorous, moderate, or light activity for at least 10 min per week, and if the respondent was physically active in one, two, or more intensities of them, the respondent was recorded as “low to moderately active” or “highly active”, respectively, and if none of them was involved, the respondent was recorded as “inactive”. Family size is divided into three categories based on the number of family members living together, including “small (1 to 2),” “medium (3 to 4),” and “large (5 or more).” We classified respondents’ family socioeconomic status as “low,” “relatively low,” “relatively high,” and “high” based on the 25th, 50th, and 75th percentiles of their total annual household expenditures. Finally, we controlled for the type of household registration of the respondents according to the reality of urban-rural differences, distinguishing between “agricultural hukou” and “non-agricultural hukou”.

#### Measurement of ICFCOA

To represent the degree of ICFCOA and for robustness considerations, we use the 90-10th interquartile range (IQ range), the 90-10th interquartile ratio (IQ ratio), the Gini coefficient (Gini), and the coefficient of variation (CV) for the calculations, respectively. Due to space limitations, the definitions of the above four indicators and the construction of their RIFs, which can be found in the studies of Firpo et al. (2018) [23], Choe and Van Kerm (2018), and Firpo and Pinto (2016) [24], are not presented further.

### Realisation of statistical and econometric analysis

In the descriptive statistical analysis section, continuous and categorical variables were described using means ± standard deviations and frequencies (%), respectively, while differences in variables within gender subgroups were compared using Student’s *t*-test and Chi-squared test. In the econometric analysis section, all models controlled for the vector set of control variables included in this study and additionally controlled for three high-dimensional fixed effects of respondents’ community of residence, current work status, and year of the interview in both the RIF-UQR and RIF-OLS models to eliminate confounding from other potential natural ecological circumstances, socioeconomic status, and temporal heterogeneity that may affect causes estimated bias. It should be noted that, following the recommendations of Firpo et al. (2009) [25] and Rios-Avila (2020) [26], we used the bootstrap method in estimating the standard errors of all models to avoid the problem of low OLS standard errors, thus obtaining more realistic and reliable *P*-value estimates. Detailed empirical strategies and main principles are available through supplementary materials. In addition, as a robustness check of the results, we used different indicators as explained variables in the RIF-OLS regression and subsequent Oaxaca-Blinder decomposition for ICFCOA. For the identification of the overall gender difference effect, we examined and compared four regressions in turn, including the inclusion of control variables with reweighting treatment, the inclusion of control variables only, the reweighting treatment only, and the inclusion of control variables without reweighting treatment, and supplemented with the propensity score matching (PSM) method to verify the existence of the overall gender difference effect. All analytical work in this study was performed using Stata MP 16.

## Results

### Identification of factors influencing CFCOA: results from RIF-UQR

**Fig. 1:**
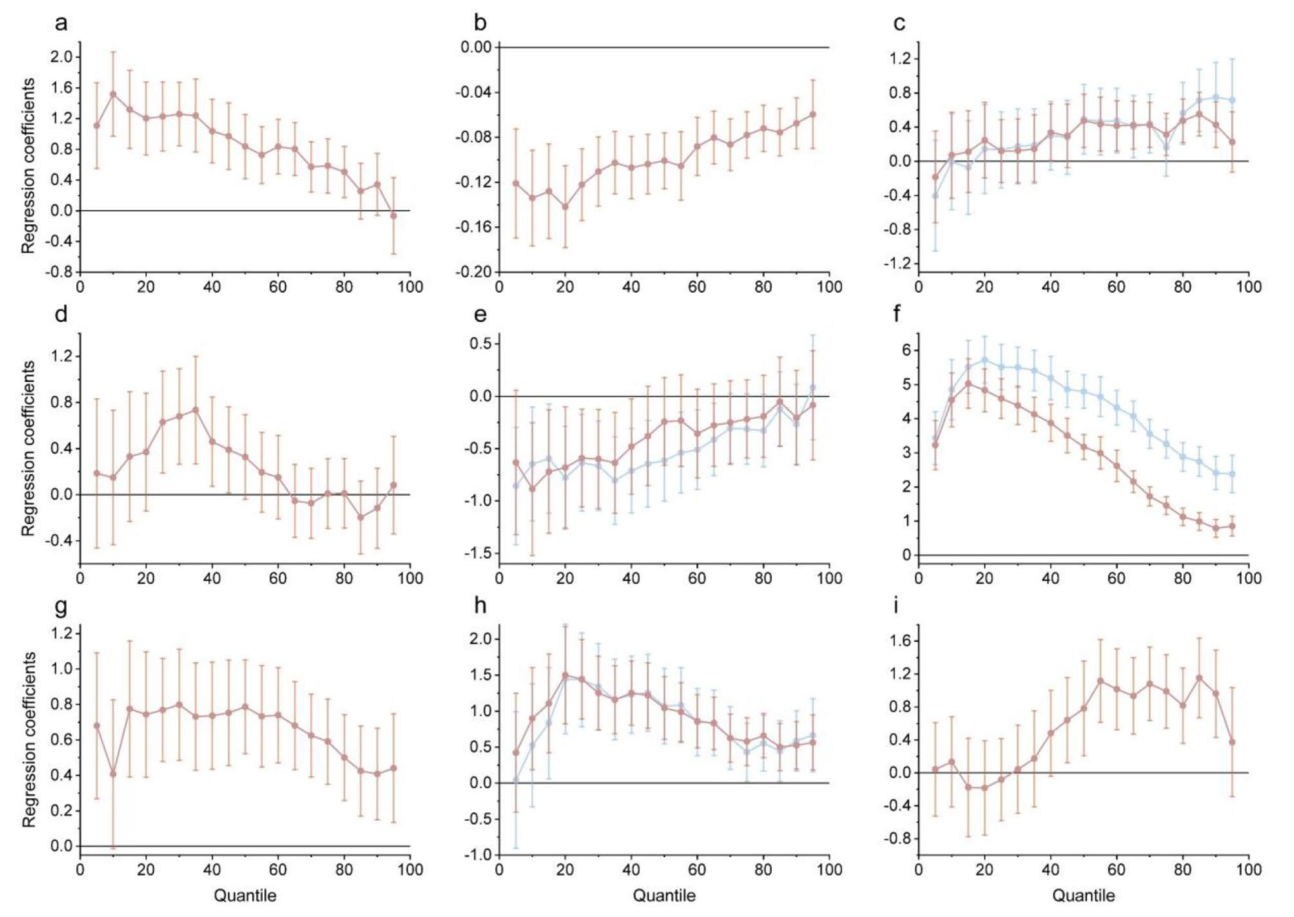
Results of RIF-QUR with gender **(a)**, age **(b)**, self-reported health **(c)**, marital status **(d)**, smoking **(e)**, educational attainment **(f)**, social interaction **(g)**, physical activity **(h)**, and type of household registration **(i)** as explained variables, respectively

As shown in Table 1, the OLS regression results indicate that gender, age, low BMI, self-reported health, smoking, educational attainment, social interaction, physical activity, and type of household registration all affect CFCOA, which is statistically significant at least at the 5% level. From models 2 to 4 in Table 1, it can be seen that all the RIF-UQR estimated results remain consistent with the OLS estimated results in terms of coefficient significance, except for the variable, low BMI. Meanwhile, to more fully show the heterogeneity information in the RIF-UQR estimated results, we plotted Fig. 1 based on the regression results of the explanatory variables with statistical significance. From Table 1; Fig. 1, elderly males had a greater advantage in cognitive function compared with elderly females, but this relative advantage showed a decreasing trend as the quartile shifted upward, and the regression coefficients were not statistically significant at the higher quartile. There was a negative effect of age on CFCOA, and the absolute value of the regression coefficient showed a fluctuating decrease as the quantile moved upward, from 0.121 at the 5th quantile to 0.060 at the 95th quantile. There was no statistically significant effect of self-reported health on CFCOA below the 50th percentile, whereas there was a statistically significant positive effect on CFCOA at the 50th percentile and above. Married older adults had a significant advantage in cognitive function compared to unmarried older adults only in the 25th to 45th quartile. Smoking, especially current or former smoking, had a negative effect on cognitive function in the low to middle quartiles, but the effect diminished and became nonsignificant as the quartile increased. An interesting finding is that there is a positive effect of education on CFCOA, shown as an inverted U-shape in Fig. 1, with this promotion effect increasing between the 5th and 15th quartiles and decreasing further beyond the 15th quartile, and higher levels of education lead to more cognitive improvement. Social interaction and physical activity have promotion effects on CFCOA and have relatively similar curves of effects. In addition, the type of household registration had no significant effect on CFCOA in the lower quartiles, while those with urban household registration in quartile 45 and above had a significant cognitive function advantage over the reference group. In contrast, the overall RIF-UQR estimation results indicated that variables such as BMI, waist circumference, alcohol consumption, family size, and family socioeconomic status did not significantly affect CFCOA.

**Table 1:** RIF-UQR regression results of the factors influencing CFCOA

Characteristics/Variables (Reference group)	Model 1^a^		Model 2^b^		Model 3^b^		Model 4^b^	
	OLS	*P*-value	Q25	*P*-value	Q50	*P*-value	Q75	*P*-value
Gender (Females)	0.793^**^	≤ 0.001	1.228^**^	≤ 0.001	0.838^**^	≤ 0.001	0.585^**^	≤ 0.001
Age	-0.097^**^	≤ 0.001	-0.122^**^	≤ 0.001	-0.101^**^	≤ 0.001	-0.078^**^	≤ 0.001
BMI (Normal: 18.5 ≤ BMI < 24)								
Light (12 ≤ BMI < 18.5)	-0.434^*^	0.027	-0.765^*^	0.046	-0.566	0.057	-0.367	0.125
Heavy (24 ≤ BMI < 28)	0.108	0.365	0.115	0.589	0.308	0.093	0.085	0.590
Obese (28 ≤ BMI ≤ 60)	0.244	0.262	0.322	0.392	0.608	0.069	0.360	0.197
Waist	0.007	0.320	0.007	0.621	-0.007	0.556	-0.006	0.515
Subjective health (Poor)								
Moderate	0.280^**^	0.005	0.121	0.521	0.475^**^	0.003	0.314^*^	0.012
Excellent	0.318^*^	0.016	0.134	0.554	0.493^*^	0.017	0.163	0.342
Marital status (Not in marriage)	0.190	0.127	0.630^**^	0.005	0.328	0.079	0.010	0.950
Smoking (Never)								
Former	-0.373^**^	0.005	-0.589^*^	0.014	-0.242	0.256	-0.217	0.252
Long-term/Current	-0.475^**^	≤ 0.001	-0.633^**^	0.007	-0.614^**^	0.002	-0.313	0.067
Drinking (Never)								
Less than once a month	0.013	0.928	0.420	0.096	0.076	0.735	-0.298	0.140
More than once a month	-0.003	0.981	0.026	0.900	-0.050	0.788	-0.035	0.822
Education (No formal education)								
Elementary school or below	2.825^**^	≤ 0.001	4.594^**^	≤ 0.001	3.174^**^	≤ 0.001	1.453^**^	≤ 0.001
Junior high school or above	4.110^**^	≤ 0.001	5.514^**^	≤ 0.001	4.798^**^	≤ 0.001	3.264^**^	≤ 0.001
Social interaction (Inactive)	0.649^**^	≤ 0.001	0.770^**^	≤ 0.001	0.787^**^	≤ 0.001	0.591^**^	≤ 0.001
Physical activity (Inactive)								
Low to moderately active	0.879^**^	≤ 0.001	1.445^**^	≤ 0.001	1.044^**^	≤ 0.001	0.578^**^	≤ 0.001
Highly active	0.817^**^	≤ 0.001	1.435^**^	≤ 0.001	1.070^**^	≤ 0.001	0.431^*^	0.036
Family size (Small: 1 to 2)								
Medium (3 to 4)	-0.076	0.460	0.093	0.613	-0.221	0.137	-0.070	0.599
Large (5 or more)	-0.097	0.418	-0.064	0.765	-0.097	0.602	-0.164	0.295
Family socioeconomic status (Low)								
Relatively low	0.028	0.829	0.295	0.219	0.109	0.595	-0.049	0.773
Relatively high	-0.242	0.088	-0.312	0.267	-0.287	0.213	-0.171	0.358
High	-0.165	0.175	-0.157	0.484	-0.104	0.598	-0.214	0.173
Hukou (Agricultural hukou)	0.540^**^	≤ 0.001	-0.082	0.746	0.783^**^	0.008	0.991^**^	≤ 0.001
Constants	12.122^**^	≤ 0.001	9.681^**^	≤ 0.001	13.244^**^	≤ 0.001	16.284^**^	≤ 0.001
Work status FE	Yes		Yes		Yes		Yes	
Community FE	Yes		Yes		Yes		Yes	
Interviewed FE	Yes		Yes		Yes		Yes	
Adjusted R-squared	0.396		0.261		0.279		0.234	
RMSE	2.673		4.855		4.113		3.522	
Average RIF	—		8.345		10.973		13.258	
Observations	4821		4821		4821		4821	

CFCOA, cognitive function in Chinese older adults; BMI, body mass index; FE: fixed effect; RMSE, Root mean squared error; RIF, recentered influence function; UQR, unconditional quantile regression; OLS, ordinary least squares.

^a^ Model 1 was estimated using OLS regression.

^b^ Models 2 to 4 were estimated using RIF-UQR.

Standard errors and *P*-values for all models were obtained using bootstrap methods with 500 replications.

^*^P < 0.05, ^**^P < 0.01.

### Identification of factors influencing ICFCOA: results from RIF-OLS

The results of the RIF-OLS regression are presented in Table 2. The mean 90-10th IQ range, 90-10th IQ ratio, Gini, and CV used to measure ICFCOA were 9.086, 2.619, 0.188, and 0.333, respectively. In contrast to the previously estimated results obtained using the RIF-UQR, after replacing the explained variables with indicators measuring ICFCOA, the number of variables showing statistical significance in the original regression coefficients shrank, with only three variables (i.e., gender, age, and educational attainment) simultaneously passing the significance test in Models 1 to 4. The results of Models 1 to 4 showed that if the proportion of elderly males in the total population of respondents increased by 10%, the absolute gap and the relative ratio of cognitive function between the top 10% of older adults and the bottom 10% of older adults, ranked by level of cognitive function, would decrease by 1.3% (1.175/9.086 × 0.1) and 2.6% (0.668/1.841 × 0.1), respectively, while the Gini and CV would decrease by 2.1% (0.040/0.188 × 0.1) and 2.0% (0.067/0.333 × 0.1), respectively. Age was a positive shock factor for the ICFCOA, with the predicted absolute gap and the relative ratio of cognitive function between the top 10% of older adults in the ranking and the bottom 10% of older adults increasing by 0.7% (0.066/9.086) and 2.0% (0.052/2.619), respectively, for each year increase in the mean age of respondents, and similar predictive results were found for the Gini and CV. In terms of educational attainment, a significant decrease in ICFCOA would occur if the proportion of respondents with primary education (or below) or medium education (or above) was increased compared to those with no formal education. In addition, other statistically significant regression results (mainly regression results for Gini and CV) suggest that smoking may exacerbate ICFCOA to some extent, while physical activity facilitates the reduction of such inequalities.

**Table 2:** RIF-OLS regression results of ICFCOA

Characteristics/Variables (Reference group)	Model 1^a^		Model 2^b^		Model 3^c^		Model 4^d^	
	IQ Range	*P*-value	IQ Ratio	*P*-value	Gini	*P*-value	CV	*P*-value
Gender (Females)	-1.175^**^	≤ 0.001	-0.668^**^	≤ 0.001	-0.040^**^	≤ 0.001	-0.067^**^	≤ 0.001
Age	0.066^**^	0.008	0.052^**^	≤ 0.001	0.003^**^	≤ 0.001	0.006^**^	≤ 0.001
BMI (Normal: 18.5 ≤ BMI < 24)								
Light (12 ≤ BMI < 18.5)	0.140	0.805	0.172	0.509	0.011	0.333	0.017	0.406
Heavy (24 ≤ BMI < 28)	-0.354	0.259	-0.141	0.262	-0.006	0.251	-0.010	0.275
Obese (28 ≤ BMI ≤ 60)	-0.009	0.987	-0.052	0.817	-0.001	0.956	0.003	0.865
Waist	-0.001	0.955	-0.007	0.389	0.000	0.382	-0.001	0.306
Subjective health (Poor)								
Moderate	0.359	0.211	0.045	0.730	0.002	0.768	0.004	0.686
Excellent	0.756^*^	0.034	0.140	0.347	0.008	0.250	0.015	0.220
Marital status (Not in marriage)	-0.267	0.436	-0.093	0.534	-0.013	0.060	-0.018	0.110
Smoking (Never)								
Former	0.683	0.097	0.390^*^	0.017	0.019^*^	0.014	0.032^*^	0.014
Long-term/Current	0.379	0.270	0.262	0.067	0.023^**^	≤ 0.001	0.040^**^	≤ 0.001
Drinking (Never)								
Less than once a month	-0.611	0.135	-0.178	0.280	-0.015^*^	0.045	-0.024	0.055
More than once a month	-0.017	0.954	0.012	0.917	-0.004	0.497	-0.006	0.543
Education (No formal education)								
Elementary school or below	-3.761^**^	≤ 0.001	-2.047^**^	≤ 0.001	-0.132^**^	≤ 0.001	-0.218^**^	≤ 0.001
Junior high school or above	-2.441^**^	≤ 0.001	-1.894^**^	≤ 0.001	-0.136^**^	≤ 0.001	-0.222^**^	≤ 0.001
Social interaction (Inactive)	0.001	0.997	-0.121	0.254	-0.019^**^	≤ 0.001	-0.033^**^	≤ 0.001
Physical activity (Inactive)								
Low to moderately active	-0.371	0.365	-0.335	0.071	-0.029^**^	≤ 0.001	-0.046^**^	0.002
Highly active	0.062	0.894	-0.145	0.488	-0.023^*^	0.016	-0.034^*^	0.045
Family size (Small: 1 to 2)								
Medium (3 to 4)	-0.247	0.380	-0.100	0.397	-0.002	0.685	-0.004	0.633
Large (5 or more)	-0.361	0.296	-0.115	0.411	-0.003	0.602	-0.007	0.535
Family socioeconomic status (Low)								
Relatively low	-0.400	0.284	-0.104	0.518	-0.008	0.283	-0.012	0.348
Relatively high	-0.198	0.615	0.033	0.843	0.005	0.479	0.009	0.471
High	-0.012	0.973	0.086	0.572	0.007	0.324	0.013	0.272
Hukou (Agricultural hukou)	0.829^*^	0.033	0.112	0.450	0.005	0.541	0.007	0.601
Constants	7.973^**^	≤ 0.001	1.841	0.092	0.141^**^	0.002	0.241^**^	0.003
Work status FE	Yes		Yes		Yes		Yes	
Community FE	Yes		Yes		Yes		Yes	
Interviewed FE	Yes		Yes		Yes		Yes	
Adjusted R-squared	0.075		0.120		0.227		0.212	
RMSE	7.217		2.999		0.140		0.241	
Average RIF	9.086		2.619		0.188		0.333	
Observations	4821		4821		4821		4821	

ICFCOA, inequalities in cognitive function in Chinese older adults; IQ range, the 90-10th interquartile range; IQ ratio, the 90-10th interquartile ratio; Gini, the Gini coefficient; CV, the coefficient of variation; BMI, body mass index; FE: fixed effect; RMSE, Root mean squared error; RIF, recentered influence function; OLS, ordinary least squares.

^a^ In Model 1, the IQ range was used as the explained variable.

^b^ In Model 2, the IQ ratio was used as the explained variable.

^c^ In Model 3, the Gini was used as the explained variable.

^d^ In Model 4, the CV was used as the explained variable.

Standard errors and *P*-values for all models were obtained using bootstrap methods with 500 replications.

^*^P < 0.05, ^**^P < 0.01.

### Gender differences in ICFCOA: estimation of effects

Four different estimation strategies are used in Models 1 to 4, as shown in Table 3, where Models 1 and 2 do not include control variables and may have endogeneity problems due to omitted variables, and the estimated results may be biased. However, for the sake of accuracy, we use Model 4, which includes all control variables and uses the reweighting adjustment method, as the final estimated result. Overall, elderly males had a mean cognitive score of 0.887 (*p* ≤ 0.001) higher than elderly females, with a larger difference at the 10th quartile ($\beta$= 0.900, *p* = 0.005) and a smaller non-significant difference at the 90th quartile ($\beta$= 0.245, *p* = 0.295). Except for the 90-10th IQ range, the regression results for the other inequality indicators showed less inequality in cognitive function and less internal variation between elderly males compared to elderly females. To further validate the reliability of the estimated sex effect of the ICFCOA, we also regressed the above model using the PSM method (Supplemental Table S6). The estimated results under the four different matching strategies were highly consistent in terms of the sign and significance of the coefficients compared with the results of the RIF effect identification, although there were slight differences in the magnitude of the coefficients, further validating the existence of the above effect.

**Table 3:** Results of gender-differentiated RIF-OLS regression of ICFCOA

Gender grouping	Model 1^a^: No controls		Model 2^b^: No controls + IPW		Model 3^c^: Controls		Model 4^d^: Controls + IPW	
	OTE 1	*P*-value	OTE 2	*P*-value	OTE 3	*P*-value	OTE 4	*P*-value
Mean	1.278^**^	≤ 0.001	1.013^**^	≤ 0.001	0.750^**^	≤ 0.001	0.887^**^	≤ 0.001
10th quantile	1.893^**^	≤ 0.001	0.919^*^	0.015	1.192^**^	≤ 0.001	0.900^**^	0.005
90th quantile	0.326^**^	0.009	0.275	0.341	0.063	0.693	0.245	0.295
90-10th IQ range	-1.566^**^	≤ 0.001	-0.640	0.187	-1.128^**^	≤ 0.001	-0.650	0.104
90-10th IQ ratio	-0.797^**^	≤ 0.001	-0.346	0.100	-0.505^**^	≤ 0.001	-0.341^*^	0.040
Gini	-0.054^**^	≤ 0.001	-0.041^**^	≤ 0.001	-0.035^**^	≤ 0.001	-0.037^**^	≤ 0.001
CV	-0.088^**^	≤ 0.001	-0.070^**^	≤ 0.001	-0.059^**^	≤ 0.001	-0.063^**^	≤ 0.001
Observations	4868		4868		4868		4868	

ICFCOA, inequalities in cognitive function in Chinese older adults; IQ range, the 90-10th interquartile range; IQ ratio, the 90-10th interquartile ratio; Gini, the Gini coefficient; CV, the coefficient of variation; RIF, recentered influence function; OLS, ordinary least squares; OTE, overall treatment effect.

^a^ In Model 1, adding no control variables.

^b^ In Model 2, adding no controls but using the reweighting adjustment (IPW).

^c^ In Model 3, adding controls as in Tables 1 and 2, but without the reweighting adjustment.

^d^ In Model 4, adding both controls and the reweighting adjustment.

Standard errors and *P*-values for all models were obtained using bootstrap methods with 500 replications.

^*^P < 0.05, ^**^P < 0.01.

### The source of gender differences in ICFCOA: results from Oaxaca-Blinder decomposition

The above work has demonstrated the existence of gender differences in ICFCOA, so an important and consequential work to be explored is to find the source of such gender differences. Therefore, this section deconstructs the source of these gender differences using the Oaxaca-Blinder decomposition method, and the results are shown in Table 4 and Supplemental Table S7. All models demonstrate the existence of gender differences in ICFCOA, i.e., this inequality is approximately 19.2–36.0% higher for females compared to males, and the coefficients of the model specification error and reweighting error terms are not statistically significant in all models, indicating that the Oaxaca-Blinder decomposition models fit well. The ICFCOA was greater for females, with 28.2–36.3% of the differences explained by individual characteristics and 51.9–71.8% of the unexplained differences. For example, in terms of the quantile range indicator, 36.3% of the ICFCOA differences come from the explainable part, while further decomposition results show that educational attainment (40.2–68.4%) and marital status (16.7%) contribute most to the purely explainable differences. However, there was also a positive contribution of unexplained differences to ICFCOA (63.7%), suggesting that factors such as education level and marital status amplify ICFCOA more for elderly females than for elderly males.

**Table 4:** Results of Oaxaca-Blinder decomposition of gender differences in ICFCOA

Gender grouping	Model 1^a^			Model 2^b^		
	IQ range	*P*-value	Percentage	IQ ratio	*P*-value	Percentage
Female group	9.706^**^	≤ 0.001	—	3.009^**^	≤ 0.001	—
Counterfactual group	8.709^**^	≤ 0.001	—	2.437^**^	≤ 0.001	—
Male group	8.140^**^	≤ 0.001	—	2.212^**^	≤ 0.001	—
Total gender difference	1.566^**^	≤ 0.001	100.0%	0.797^**^	≤ 0.001	100.0%
Total characteristic effect	0.569	0.064	36.3%	0.225^*^	0.017	28.2%
Pure characteristic effect	1.065^**^	0.003	68.0%	0.372^**^	0.002	46.7%
Model specification error	-0.496	0.163	-31.7%	-0.147	0.188	-18.4%
Total coefficient effect	0.997^**^	0.003	63.7%	0.572^**^	≤ 0.001	71.8%
Pure coefficient effect	0.982^**^	0.004	62.7%	0.555^**^	≤ 0.001	69.6%
Reweighting error	0.015	0.906	1.0%	0.017	0.683	2.1%

ICFCOA, inequalities in cognitive function in Chinese older adults; IQ range, the 90-10th interquartile range; IQ ratio, the 90-10th interquartile ratio.

^a^ In Model 1, the IQ range was used as the explained variable.

^b^ In Model 2, the IQ ratio was used as the explained variable.

Standard errors and *P*-values for all models were obtained using bootstrap methods with 500 replications.

^*^P < 0.05, ^**^P < 0.01.

## Discussion

To the best of our knowledge, although existing studies have paid attention to the factors influencing CFCOA and its gender differences, the relevant empirical methods and perspectives are quite homogeneous, and the widespread use of mean regression does not allow for obtaining more potentially useful information. In addition, studies have yet to be conducted to explore ICFCOA, and the related derivative questions have received even less attention. In contrast, using the CHARLS dataset from 2011 to 2015, this study is the first to introduce a set of econometric methods based on RIFs for identifying the influencing factors, estimating the gender difference effects, and decomposing them for ICFCOA, attempting to fill the gap of relevant studies in the academic community. In this study, we also obtained some interesting findings after a more rigorous and standardised quantitative analysis. These new insights are crucial to facilitate the government and society to identify and practically adopt interventions that can help mitigate cognitive deterioration and ICFCOA.

The results of the RIF-UQR regression showed that elderly males had higher levels of cognitive function than elderly females, which is consistent with the findings of existing studies [27] and may be closely related to physiological characteristics, socioeconomic status, and life course between males and females [28]. Moreover, this gender difference is very pronounced in the low cognitive function group and diminishes in the high cognitive function group, which may be explained by the inherent advantages of individual genetic and biological factors that contribute to the generally high threshold level of human cognitive ability in some males and females and its persistence over time [29, 30]. Alternatively, older individuals with high cognitive function tend to have access to more similar educational opportunities and quality so that the cognitive benefits of education converge for different individuals of both sexes [31]. Age is a risk factor for cognitive decline in older adults, which many studies have confirmed [32–34], but the inherent difference in cognitive function and its rate of decline between older adults with high cognitive function and those with low cognitive function seems to suggest that there is “Matthew effect” on CFCOA. From the results of BMI and waist circumference analysis, only BMI that was low in a few quartiles showed a negative effect on CFCOA, and the rest of the regression results for BMI and waist circumference were not statistically significant. Some previous studies have found that higher BMI [22] and waist circumference [22] are protective factors against cognitive decline in older adults, while others have found the opposite [35–37]. Our study supports some of these findings [35] but confirms that there is no direct relationship between BMI, waist circumference, and CFCOA. In our opinion, the apparently conflicting findings of existing studies are likely spurious associations due to omitting important explanatory variables. On average, the OLS regression results indicated a positive relationship between self-reported health and CFCOA [38], whereas the RIF-UQR regression results indicated that this relationship was statistically significant only for older adults with higher cognitive function. This finding is likely due to the use of self-reported health as a measure of the health status of older adults. Although the corresponding survey question was worded in a simple manner, it requires respondents to cognitively process multiple health information, which is difficult for older adults with poor cognitive function, i.e., they have cognitive ambiguity about their health status, which may lead to potential reporting bias [39, 40]. In contrast, such problems are rare among older adults with higher levels of cognition. Existing studies have shown that marital status can have a significant effect on CFCOA [41–43], and we found that being in marriage has a significant promotion effect in older adults with lower-middle levels of cognitive function, which partially confirms the views of existing studies. Smoking is detrimental to CFCOA, especially to low-middle cognitive function in older adults. Older adults with high cognitive function have a strong sense of self-management and the ability to reduce and control the frequency of smoking as much as possible in their later years to mitigate the adverse effects of smoking on their health. Consistent with some extensive literature, education has a positive effect on CFCOA, and the effect is more pronounced in those with higher levels of education [44, 45]. Interestingly, we also found an “inverted U-shape” in this promotion effect of education, i.e., the cognitive promotion effect of education was significantly higher in older adults with low to middle levels of cognitive function than in those with high levels of cognitive function. Both social interaction and physical activity have positive effects on CFCOA, which can maintain and promote CFCOA and reduce the rate and extent of cognitive decline in various ways, including improving the physical function and emotional status of older adults [46, 47]. At the same time, CFCOA did not show a significant urban-rural difference at the low and middle levels of cognitive function, while at the high and middle levels of cognitive function, urban older adults had significantly better cognitive function than rural older adults, which was mainly due to the fact that individuals with cognitive advantages in their younger years moved from rural to urban areas in search of higher quality study and work opportunities and maintained or even strengthened their cognitive abilities, which correspondingly widened the difference in cognitive function between urban and rural older adults in their later years [48, 49]. In terms of other non-significant variables, low frequency of alcohol consumption did not increase the risk of cognitive decline in older adults and also did not have a significant promotion effect on CFCOA [50]. It should be noted that this study was unable to assess the effect of high alcohol consumption on CFCOA because few individuals with alcohol abuse were identified in the survey. The effects of household size and household economic status on CFCOA are not statistically significant, but the signs of the coefficients are generally negative. A reasonable explanation may be that in their younger years, respondents face more work pressure and perform more mental or physical labour in the process of raising and educating their children, working, and accumulating wealth, which may be exchanged to some extent for better family living conditions in their later years, but also accelerate the depreciation of their health capital [51]. Especially in their old age, the traditional family structure, values and intergenerational relations have changed profoundly due to the changing times, and the protective and supportive functions of the family have weakened [52, 53]. In this case, the high contribution of older adults in the early stage of life has not been rewarded with adequate old-age pensions, which may adversely affect the cognitive function of older adults.

In the influencing factor analysis section for ICFCOA, we found that in addition to gender, age, education, smoking, and physical activity had significant effects on ICFCOA. In particular, increasing age will worsen ICFCOA in the full sample, and there is great heterogeneity in the trajectory of cognitive decline among elderly individuals [54], with different shapes of the cognitive curve, and the differences in cognitive function among elderly individuals will subsequently widen. Educational attainment is an important factor in narrowing the ICFCOA, i.e., education makes a significant contribution to cognitive function in the general population [44], while the same level of education has a relatively smaller marginal boost for older adults with high cognitive function. As mentioned in the previous discussion, smoking is more likely to cause cognitive impairment in older adults with low to middle cognitive function, while older adults with high cognitive function are minimally affected by smoking, and this difference in effect subsequently exacerbates the divergence in cognitive function between smokers and nonsmokers. In addition, physical activity, similar to the role of education, has a weak inverted U-shaped feature on cognitive facilitation in older adults, i.e., physical activity has a greater facilitating effect on older adults with low cognitive function compared to those with high cognitive function.

From the previous analyses, it is clear that gender is an important factor affecting CFCOA and ICFCOA, and after rigorous treatment effects analysis, we still confirm the existence of this gender difference. Based on this, we went through the Oaxaca-Blinder decomposition to further identify the main sources of this gender difference. Regarding characteristic effects, male older adults are significantly more likely to be in marriage and have higher educational attainment than female older adults. Perhaps the reason why more males are in marriage than females is that females have a significant advantage over males in terms of longevity due to biological and life factors [55–57], and therefore males can maintain their marriages longer and receive more stable support and care from their spouses [58]. This phenomenon is also visually evident in the descriptive statistics of the total sample, i.e., 87.6% of the older male samples were in marriages, while only 76.8% of the older female samples were in marriages. In addition, we found a dual effect of education on ICFCOA, one of which was a shrinking effect of the total population differences in cognitive function, which was confirmed by our previous analysis, and the other was an increasing effect of the cognitive function differences in the sex-specific population found in this section. In the last century, China’s educational resources were scarce and educational inequality was a serious problem [59]. Under the influence of scarce educational resources, traditional family values, and gender discrimination, male offspring were more likely to have access to education than female offspring and could improve their cognitive abilities and increase their relative cognitive advantages through education [60]. Similarly, unexplained differences significantly positively contributed to the overall difference, as factors such as marital status and educational attainment had a smaller shrinking effect on ICFCOA for females than for males.

The above findings have some practical implications for the government and society to take comprehensive initiatives to maintain and improve CFCOA, gradually narrow the cognitive function gap, reduce the probability of cognitive decline, and delay the age of onset of cognitive decline in older adults. In promoting the “Healthy China” strategy and ageing work in the new era, the potential burden and real challenges of ICFCOA to the sustainable healthy development of families, economy and society should be fully appreciated. Regular assessment and follow-up of CFCOA by family doctors and primary healthcare institutions can provide reliable monitoring data for early intervention for older adults with or at risk of cognitive decline. It is necessary to accurately identify the specific effects of various physiological and socioeconomic factors on older adults in different quartiles of cognitive function, and start from the aspects of marriage maintenance, re-education in old age, living habits, social interaction and physical activities according to the actual situation, and guide older adults to establish optimistic concepts of ageing and form a healthy lifestyle suitable for themselves. Meanwhile, we should further improve the infrastructure for older adults and build an age-friendly society; for example, we can try to establish platforms such as “senior academies” or “senior recreation centers” to provide good infrastructure and interactive space for older adults to do something interesting, including continuous learning, social interaction and physical activities.

### Strengths and limitations

To the best of our knowledge, this study represents the first investigation into cognitive function among Chinese older adults (CFCOA) and inequalities in cognitive function in Chinese older adults (ICFCOA). This investigation utilizes data from a population-based cohort and employs advanced quantitative research methods, thereby ensuring the reliability and robustness of the findings. The outcomes of this study provide a valuable reference for the academic community to reassess CFCOA and ICFCOA, as well as for policymakers to advance related work. Nevertheless, it is worth noting that this investigation did not take into account the dynamic changes of CFCOA and ICFCOA, which could be a promising avenue for future research by introducing time variables to the analysis. Therefore, future research can further explore the longitudinal changes of CFCOA and ICFCOA, in order to provide deeper insights into the trajectory of cognitive function among older adults in China.

## Conclusions

In summary, this study has some advantages and marginal contributions compared to existing studies. We introduced the first RIFs-based econometric approach in the field of CFCOA assessment based on a large nationally representative data sample and specifically did the following: (1) Identifying and analysing the effects of different factors on the heterogeneity of CFCOA at different levels; (2) Measuring the extent of ICFCOA and identifying the relevant influencing factors; (3) Confirming the existence of gender differences in ICFCOA and analysing the main sources of influence on such gender differences. This series of work can provide some reference for the academic community to re-evaluate CFCOA and the policy community to promote related work.

### Electronic supplementary material

Below is the link to the electronic supplementary material.

Supplementary Material 1

## Data Availability

The data used in this study were all available on the CHARLS public website (https://charls.pku.edu.cn/).

## Abbreviations

CFCOA: cognitive function in Chinese older adults
ICFCOA: inequalities in cognitive function in Chinese older adults
BMI: body mass index
FE: fixed effect
RMSE: Root mean squared error
RIF: recentered influence function
UQR: unconditional quantile regression
OLS: ordinary least squares
IQ range: the 90-10th interquartile range
IQ ratio: the 90-10th interquartile ratio
Gini: the Gini coefficient
CV: the coefficient of variation
OTE: overall treatment effect

## References

[1] Jensen N, Kelly AH, Avendano M (2022). Health equity and health system strengthening - time for a WHO re-think. Glob Public Health.

[2] van den Beld AW, Kaufman JM, Zillikens MC, Lamberts SWJ, Egan JM, van der Lely AJ (2018). The physiology of endocrine systems with ageing. Lancet Diabetes Endocrinol.

[3] Beard JR, Officer A, de Carvalho IA, Sadana R, Pot AM, Michel JP (2016). The World report on ageing and health: a policy framework for healthy ageing. Lancet.

[4] Coll-Planas L, Blancafort S, Rojano X, Roque M, Monteserin R (2018). Promoting self-management, health literacy and social capital to reduce health inequalities in older adults living in urban disadvantaged areas: protocol of the randomised controlled trial AEQUALIS. BMC Public Health.

[5] Fiocco AJ, Yaffe K (2010). Defining successful aging: the importance of including cognitive function over time. Arch Neurol.

[6] Xiao Y, Jia S, Zhao W, Zhang Y, Qiao R, Xia X (2021). The combined effect of hearing impairment and cognitive impairment with Health-related outcomes in Chinese older people. J Nutr Health Aging.

[7] Lara E, Koyanagi A, Caballero F, Domenech-Abella J, Miret M, Olaya B (2017). Cognitive reserve is associated with quality of life: a population-based study. Exp Gerontol.

[8] Chen R, Xu P, Song PP, Wang MF, He JJ (2019). China has faster pace than Japan in population aging in next 25 years. Biosci Trends.

[9] Liu XC, Zhu JN, Zou K (2022). The development trend of China’s aging population: a forecast perspective. Complex Intell Syst.

[10] Jia LF, Du YF, Chu L, Zhang ZJ, Li FY, Lyu DY (2020). Prevalence, risk factors, and management of dementia and mild cognitive impairment in adults aged 60 years or older in China: a cross-sectional study. Lancet Public Health.

[11] Zhang Y, Guan YL, Shi ZH, Yue W, Liu S, Liu SL (2019). Sex differences in the prevalence of and risk factors for cognitive impairment no dementia among the Elderly in a rural area of Northern China: a Population-based cross-sectional study. Neuroepidemiology.

[12] Ye X, Zhu DW, He P (2021). Earlier migration, better cognition? The role of urbanization in bridging the urban-rural cognition gaps. Int J Epidemiol.

[13] Gao YL, Liu XN (2021). Secular trends in the incidence of and Mortality due to Alzheimer’s Disease and other forms of dementia in China from 1990 to 2019: an age-period-cohort study and Joinpoint Analysis. Front Aging Neurosci.

[14] Zeng Y, Feng QS, Hesketh T, Christensen K, Vaupel JW (2017). Survival, disabilities in activities of daily living, and physical and cognitive functioning among the oldest-old in China: a cohort study. Lancet.

[15] Jin YZ, Jing MX, Ma XC (2019). Effects of Digital device ownership on Cognitive decline in a Chinese middle-aged and Elderly Population: longitudinal observational study. J Med Internet Res.

[16] Luo YN, Zhong YJ, Pang LH, Zhao YH, Liang R, Zheng XY (2021). The effects of indoor air pollution from solid fuel use on cognitive function among middle-aged and older population in China. Sci Total Environ.

[17] Pan Y (2020). Late-life cognition: do childhood conditions play any role?. China Econ Rev.

[18] Pan X, Luo Y, Roberts AR (2018). Secondhand smoke and women’s cognitive function in China. Am J Epidemiol.

[19] Chen HY, Chen L, Hao G (2021). Sex difference in the association between solid fuel use and cognitive function in rural China. Environ Res.

[20] Frolich J, Palm CVB, Stoving RK (2016). To the limit of extreme malnutrition. Nutrition.

[21] Thereaux J, Czernichow S, Corigliano N, Poitou C, Oppert JM, Bouillot JL (2015). Five-year outcomes of gastric bypass for super-super-obesity (BMI > = 60 kg/m(2)): a case matched study. Surg Obes Relat Dis.

[22] Liang F, Fu JL, Moore JB, Zhang XE, Xu YJ, Qiu N (2022). Body Mass Index, Waist circumference, and Cognitive decline among Chinese older adults: a Nationwide Retrospective Cohort Study. Front Aging Neurosci.

[23] Firpo SP, Fortin NM, Lemieux T (2018). Decomposing wage distributions using recentered influence function regressions. Econometrics.

[24] Firpo S, Pinto C (2016). Identification and estimation of distributional impacts of interventions using changes in Inequality measures. J Appl Econom.

[25] Firpo S, Fortin NM, Lemieux T (2009). Unconditional Quantile Regressions Econometrica.

[26] Rios-Avila F (2020). Recentered influence functions (RIFs) in Stata: RIF regression and RIF decomposition. Stata J.

[27] Yin JM, Lassale C, Steptoe A, Cadar D (2019). Exploring the bidirectional associations between loneliness and cognitive functioning over 10 years: the English longitudinal study of ageing. Int J Epidemiol.

[28] Subramaniapillai S, Almey A, Rajah MN, Einstein G (2021). Sex and gender differences in cognitive and brain reserve: implications for Alzheimer’s disease in women. Front Neuroendocrinol.

[29] Huguet G, Schramm C, Douard E, Tamer P, Main A, Monin P (2021). Genome-wide analysis of gene dosage in 24,092 individuals estimates that 10,000 genes modulate cognitive ability. Mol Psychiatry.

[30] Mustafin RN, Kazantseva AV, Malykh SB, Khusnutdinova EK (2020). Genetic mechanisms of Cognitive Development. Russ J Genet.

[31] Herd P, Freese J, Sicinski K, Domingue BW, Harris KM, Wei CP (2019). Genes, gender inequality, and Educational Attainment. Am Sociol Rev.

[32] Ahrenfeldt LJ, Lindahl-Jacobsen R, Rizzi S, Thinggaard M, Christensen K, Vaupel JW (2018). Comparison of cognitive and physical functioning of europeans in 2004-05 and 2013. Int J Epidemiol.

[33] Atalay K, Barrett GF, Staneya A (2019). The effect of retirement on elderly cognitive functioning. J Health Econ.

[34] Park DC, Polk TA, Mikels JA, Taylor SF, Marshuetz C. Cerebral aging: integration of brain and behavioral models of cognitive function. Dialogues Clin Neurosci. 2022. 10.31887/DCNS.2001.3.3/dcpark PMC3181659 22034448

[35] Kivimaki M, Luukkonen R, Batty GD, Ferrie JE, Pentti J, Nyberg ST (2018). Body mass index and risk of dementia: analysis of individual-level data from 1.3 million individuals. Alzheimers Dement.

[36] Liu ZZ, Yang HQ, Chen SY, Cai J, Huang ZJ (2019). The association between body mass index, waist circumference, waist-hip ratio and cognitive disorder in older adults. J Public Health.

[37] West NA, Lirette ST, Cannon VA, Turner ST, Mosley TH, Windham BG, Adiposity (2017). Change in Adiposity, and Cognitive decline in Mid- and late life. J Am Geriatr Soc.

[38] Zaninotto P, Batty GD, Allerhand M, Deary IJ (2018). Cognitive function trajectories and their determinants in older people: 8 years of follow-up in the English Longitudinal Study of Ageing. J Epidemiol Community Health.

[39] Bollen KA, Gutin I, Halpern CT, Harris KM (2021). Subjective health in adolescence: comparing the reliability of contemporaneous, retrospective, and proxy reports of overall health. Soc Sci Res.

[40] Lokshin M, Ravallion M (2008). Testing for an economic gradient in health status using subjective data. Health Econ.

[41] Chen ZC, Wu H, Wang XD, Zeng Y, Huang GW, Lv Y (2022). Association between marital status and cognitive impairment based on a cross-sectional study in China. Int J Geriatr Psychiatry.

[42] Shen S, Cheng JD, Li JP, Xie YY, Wang L, Zhou XL (2022). Association of marital status with cognitive function in Chinese hypertensive patients: a cross-sectional study. BMC Psychiatry.

[43] Xu PR, Wei R, Cheng BJ, Wang AJ, Li XD, Li HB (2021). The association of marital status with cognitive function and the role of gender in Chinese community-dwelling older adults: a cross-sectional study. Aging Clin Exp Res.

[44] Lovden M, Fratiglioni L, Glymour MM, Lindenberger U, Tucker-Drob EM (2020). Education and cognitive functioning across the Life Span. Psychol Sci Public Interest.

[45] Park S, Choi B, Choi C, Kang JM, Lee JY (2019). Relationship between education, leisure activities, and cognitive functions in older adults. Aging Ment Health.

[46] Elovainio M, Sommerlad A, Hakulinen C, Pulkki-Raback L, Virtanen M, Kivimaki M (2018). Structural social relations and cognitive ageing trajectories: evidence from the Whitehall II cohort study. Int J Epidemiol.

[47] Kuiper JS, Zuidersma M, Zuidema SU, Burgerhof JGM, Stolk RP, Voshaar RCO (2016). Social relationships and cognitive decline: a systematic review and meta-analysis of longitudinal cohort studies. Int J Epidemiol.

[48] Chuang YF, Liu YC, Tseng HY, Lin PX, Li CY, Shih MH (2021). Urban-rural differences in the prevalence and correlates of mild cognitive impairment in community-dwelling older adults in Taiwan: the EMCIT study. J Formos Med Assoc.

[49] Xu HZ, Dupre ME, Ostbye T, Vorderstrasse AA, Wu B (2019). Residential mobility and cognitive function among Middle-aged and older adults in China. Res Aging.

[50] Hassing LB (2018). Light alcohol consumption does not protect cognitive function: a longitudinal prospective study. Front Aging Neurosci.

[51] de Souza-Talarico JN, Suemoto CK, Santos IS, Griep RH, Yamaguti STF, Lotufo PA (2020). Work-related stress and cognitive performance among middle-aged adults: the Brazilian Longitudinal Study of Adult Health (ELSA-Brasil). Stress Health.

[52] Fowler AR, Gao J, Carlson L (2010). Public Policy and the changing Chinese Family in Contemporary China: the past and present as Prologue for the future. J Macromarketing.

[53] Song J, Ji YC (2020). Complexity of Chinese Family Life: Individualism, Familism, and gender. China Rev.

[54] Tu LH, Lv XZ, Yuan CZ, Zhang M, Fan ZL, Xu XL (2020). Trajectories of cognitive function and their determinants in older people: 12 years of follow-up in the Chinese longitudinal healthy longevity survey. Int Psychogeriatr.

[55] Eskes T, Haanen C (2007). Why do women live longer than men?. Eur J Obstet Gynecol Reproductive Biology.

[56] Ongel ME, Yildiz C, Akpinaroglu C, Yilmaz B, Ozilgen M (2021). Why women may live longer than men do? A telomere-length regulated and diet-based entropic assessment. Clin Nutr.

[57] Zarulli V, Jones JAB, Oksuzyan A, Lindahl-Jacobsen R, Christensen K, Vaupel JW (2018). Women live longer than men even during severe famines and epidemics. Proc Natl Acad Sci U S A.

[58] Gerritsen L, Wang HX, Reynolds CA, Fratiglioni L, Gatz M, Pedersen NL (2017). Influence of negative life events and Widowhood on Risk for Dementia. Am J Geriatr Psychiatry.

[59] Guo LJ, Huang JS, Zhang Y (2019). Education Development in China: Education Return, Quality, and equity. Sustainability.

[60] Zhang HF (2017). Opportunity or new poverty trap: rural-urban education disparity and internal migration in China. China Econ Rev.

