# Supplementary material for: Influencing factors, gender differences and the decomposition of inequalities in cognitive function in Chinese older adults: a population-based cohort study
Source: BMC Geriatr. 2024 Apr 25;24:371. doi: 10.1186/s12877-024-04857-x (PMC11045435; doi:10.1186/s12877-024-04857-x)
Supplement: Supplementary file 1 — Supplementary Material 1 [file 12877_2024_4857_MOESM1_ESM.docx]

**Influencing factors, gender differences and the decomposition of inequalities in cognitive function in Chinese older adults: a population-based cohort study**

Supplemental text 1: Literature review

Supplemental text 2: Empirical strategy and main principles

Supplemental table 1: RIF-UQR regression results of the factors influencing CFCOA

Supplemental table 2: Results of gender-differentiated RIF-OLS regression of ICFCOA (Controls + IPW)

Supplemental table 3: Results of gender-differentiated RIF-OLS regression of ICFCOA (Controls)

Supplemental table 4: Results of gender-differentiated RIF-OLS regression of ICFCOA (No controls + IPW)

Supplemental table 5: Results of gender-differentiated RIF-OLS regression of ICFCOA (No controls)

Supplemental table 6: Results of gender-differentiated PSM regression of ICFCOA

Supplemental table 7: Results of Oaxaca-Blinder decomposition of gender differences in ICFCOA

**Literature review**

Changes in CFOA are a life-cycle phenomenon and a dynamic evolutionary process influenced by a combination of factors from multiple dimensions ^1^. In this case, the decline in CFOA may be due to normal physiological ageing, which generally progresses slowly and is more prevalent ^2^. In contrast, rapid and dramatic cognitive decline is likely to mean that an older individual is in a period of irreversible cognitive impairment, which, if it progresses to a stage such as dementia, could lead to a significant decline in their quality of life, as well as significant medical and care costs ^3 4^. Given the importance of healthy and intact CFOA to maintain good individual living conditions in later life and alleviate the ageing burden on families and society, the many factors that potentially affect CFOA have been extensively explored in the academic community. Some studies have indicated that physiological factors, including individual age ^5 6^, gender ^7^, BMI ^8-10^, waist circumference ^8 9^, and lifestyle factors such as marital status ^11 12^, smoking ^13-15^, and alcohol consumption ^16^, all may impact CFOA. Furthermore, some scholars have also studied from a more macroscopic perspective, suggesting that in addition to being constrained by their intrinsic factors, other socioeconomic factors, including differences in educational attainments ^17 18^, social interactions ^19 20^, physical activities ^21 22^, and socioeconomic status ^23 24^, also contribute to the heterogeneous performance of CFOA. Meanwhile, the effect of the institutional variable, urban-rural household registration differences, on CFOA (i.e., CFCOA) has received some attention in selected studies based on Chinese survey data ^25 26^.

However, it is worth noting that the studies that have been conducted have focused more on measuring the level of CFCOA and identifying the relevant influencing factors ^27-31^, and different kinds of literature have given widely divergent and even contradictory insights into the role of some of these factors. In contrast, few studies have focused on exploring and analysing the degree of cognitive inequality and its underlying correlates ^32 33^, especially in Chinese elderly samples. Although some studies have attempted to examine some of the individual characteristics and socioeconomic factors that may influence ICFCOA from an inequality perspective ^28 33 34^, unfortunately, none of these studies has used scientifically rigorous indicators of cognitive inequality, and the identification and analysis of the relevant influencing factors are significantly inadequate to provide reliable empirical evidence.

Furthermore, among the many factors that potentially affect CFCOA, gender can be included in many studies with similar findings, which directly indicates that there are clear gender differences in CFCOA, with elderly males generally having better cognitive function than elderly females ^7 35 36^. Although the current literature has made some effort to confirm the existence of ICFCOA, little has been done to identify ICFCOA within gender-specific groups and the underlying factors that contribute to such inequalities in cognitive function. The gender-specific groups of Chinese elderly have multiple heterogeneous characteristics, such as biological or socioeconomic aspects, which has become an important academic concern to identify the inequalities in cognitive function from between-group and within-subgroup perspectives to analyse the multidimensional factors behind such inequalities.

Therefore, this study uses data from the China Health and Retirement Longitudinal Study (CHARLS) for three periods from 2011 to 2015. First, using recentered influence function-unconditional quantile regression (RIF-UQR) method, we explore the distribution of cognitive function and its influencing factors in the Chinese elderly sample. Second, we use various inequality indicators to scientifically measure CFCOA and combine them with recentered influence function-ordinary least squares (RIF-OLS) regressions to identify potential influencing factors. Third, we focus our inequality analysis on the gender dimension to explore differences in the distribution of ICFCOA. Finally, we also developed an Oaxaca-Blinder decomposition of ICFCOA by gender to specifically analyse the contribution of factors behind gender differences in ICFCOA. A marginal contribution of this study is to add to the existing literature on the knowledge of CFCOA and ICFCOA and the determinants behind it, to provide a more in-depth analysis of the specific manifestations of gender differences in the above issues, and to provide some important evidence on the identification of possible sources of effects, which can provide empirical references and academic support for academic research institutes and public policy departments to find more targeted interventions to maintain cognitive function and reduce cognitive inequality among older adults.

**Empirical strategy and main principles**

**Unconditional quantile regression based on recentered influence function (RIF-UQR)**

In general, quantile regression (QR) is divided into two types: conditional quantile regression (CQR) and unconditional quantile regression (UQR). The former, proposed by Koenker and Bassett (1978), allows to robustly estimate the heterogeneous effects of explanatory variables on the explained variables at different conditional quartiles of the disturbance term.^37^ However, interpreting CQR results often requires relying on too many individual characteristics that may differ from what the researcher is interested in. To address this issue, Firpo et al. (2009) extended CQR by proposing a new UQR regression method^38^ based on the recentered influence function (RIF) that is a tool for analyzing the robustness of distribution statistics to outliers and allowing the statistical inference^39 40^, which can be used to estimate the unconditional local effects of potentially small changes in the explanatory variables on the distribution statistics. Therefore, we use a more econometrically advantageous approach, the RIF-UQR, to develop the analysis. As defined by Firpo et al. (2009)^38^, the expression for the specific RIF is as follows:

$$\begin{aligned} RIF\left\{ y_{i},v\left( F_{Y} \right) \right\}=v\left( F_{Y} \right)+IF\left\{ y_{i},v\left( F_{Y} \right) \right\}\#\left( 1 \right) \end{aligned}$$

$F_{Y}$ is the original distribution function, and it is easy to see from equation (1) that $RIF$ consists of two parts: the distribution statistic $v\left( F_{Y} \right)$ and the influence function $IF\left\{ y_{i},v\left( F_{Y} \right) \right\}$.

$$\begin{aligned} dF_{Y}\left( y_{i} \right)=\int dF_{Y|X}\left( y_{i} | \mathbf{X}=\mathbf{x} \right)dF_{X}\left( \mathbf{x} \right)\#\left( 2 \right) \end{aligned}$$

$$\begin{aligned} F_{Y}\left( y_{i} \right)=\int F_{Y|X}\left( y_{i} | \mathbf{X}=\mathbf{x} \right)dF_{X}\left( \mathbf{x} \right)\#\left( 3 \right) \end{aligned}$$

Suppose $dF_{Y,X}\left( y,\mathbf{x} \right)=dF_{Y|X}\left( y | \mathbf{X}=\mathbf{x} \right)dF_{X}\left( \mathbf{x} \right)$ is a joint distribution function that determines the existence of a linear or nonlinear relationship between the explained variable $Y$ and all the explanatory variables or exogenous variables vector $\mathbf{X}$. Equations (1) and (2) are the cumulative distribution function (CDF) and the probability distribution function (PDF) of $Y$, respectively. Assuming that the vector $F_{Y|X}\left( y | \mathbf{x} \right)$ remains unchanged and the distribution of $\mathbf{X}$ changes from $F_{X}$ to $G_{X}$, the unconditional distribution of $Y$ changes to the following equation.

$$\begin{aligned} G_{Y}=\int F_{Y|X}\left( y_{i} | \mathbf{X}=\mathbf{x} \right)dG_{X}\left( \mathbf{x} \right)\#\left( 4 \right) \end{aligned}$$

Since the mathematical expectation of the influence function $IF\left\{ y_{i},v\left( F_{Y} \right) \right\}$ constructed using all $y_{i}$ is 0^38^, it is known that the mathematical expectation of RIF is equal to the distribution statistic itself $v\left( F_{Y} \right)$. Therefore, combining equations (2) and (3), the distribution statistic $v\left( F_{Y} \right)$ can again be rewritten as:

$$\begin{aligned} v\left( F_{Y} \right)=\iint RIF\left\{ y,v\left( F_{Y} \right) \right\}dF_{y|X}\left( y | \mathbf{X}=\mathbf{x} \right)dF_{X}\left( \mathbf{x} \right)\#\left( 5 \right) \end{aligned}$$

$$\begin{aligned} v\left( F_{Y} \right)=\int E\left[ RIF\left\{ y,v\left( F_{Y} \right) \right\} | \mathbf{X}=\mathbf{x} \right]dF_{X}\left( \mathbf{x} \right)\#\left( 6 \right) \end{aligned}$$

Combining equation (4), Firpo et al. (2009) ^38^ deduce that when the distribution of $\mathbf{X}$ changes from $F_{X}$ to $G_{X}$, equation (6) can be equivalently rewritten as:

$$\begin{aligned} v(G_{Y})-v(F_{Y})=\int E\left[ RIF\left\{ y,v\left( F_{Y} \right) \right\} | \mathbf{X}=\mathbf{x} \right]d{(G_{Y}-F}_{X})(\mathbf{X})\#\left( 7 \right) \end{aligned}$$

Suppose $E\left[ RIF\left\{ y,v\left( F_{Y} \right) \right\} | \mathbf{X}=\mathbf{x} \right]$ is a distribution function of $\mathbf{X}$. In this case, the estimated results of equations (6) and (7) are used to determine how changes in the distribution of $\mathbf{X}$ effect the distribution statistic $v\left( F_{Y} \right)$. According to Firpo et al. (2009) ^38^, the simplest way to estimate the RIF-UQR is to assume a linear relationship between $RIF\left\{ y,v\left( F_{Y} \right) \right\}$ and the explanatory variable $\mathbf{X}$. Under this assumption, OLS can be used to capture the effect of small changes in the distribution of the explanatory variable $\mathbf{X}$ on $v\left( F_{Y} \right)$.

$$\begin{aligned} RIF\left\{ y,v\left( F_{Y} \right) \right\}=\mathbf{X}^{'}\beta+\varepsilon_{i} , E\left( \varepsilon_{i} \right)=0\#\left( 8 \right) \end{aligned}$$

The difference between the above equation (8) and the standard OLS model is that RIF-OLS takes the $RIF\left\{ y,v\left( F_{Y} \right) \right\}$ of each observation, $y_{i}$, in the data as the explained variables and regresses them on all the explanatory variables of interest $\mathbf{X}$. Here, in our study, the distribution statistic of interest is the *p*th quantile, whose RIF expression was derived by Firpo et al. (2009) ^38^ as follows:

$$\begin{aligned} RIF\left\{ y,q_{Y}\left( p \right) \right\}=q_{Y}\left( p \right)+\frac{p-I\left( y\leq q_{Y}\left( p \right) \right)}{f\left\{ q_{Y}\left( p \right) \right\}}\#\left( 9 \right) \end{aligned}$$

Where $q_{Y}\left( p \right)$ is the quantile statistic of the *p*th quantile, $I\left( \cdot\right)$ is the indicator function, and $f\left\{ q_{Y}\left( p \right) \right\}$ is the marginal density function. We choose the 5th to 95th quantile as the corresponding distribution statistic to regress the vector of explanatory variables to more fully demonstrate the unconditional local effects of the vector of explanatory variables on the different quantile points of the explained variables.

**Measurement of ICFCOA**

To represent the degree of ICFCOA and for robustness considerations, we use the 90-10th interquartile range (IQ range), the 90-10th interquartile ratio (IQ ratio), the Gini coefficient (Gini), and the coefficient of variation (CV) for the calculations, respectively. Due to space limitations, the definitions of the above four indicators and the construction of their RIFs, which can be found in the studies of Firpo et al. (2018) ^41^, Choe and Van Kerm (2018) ^42^, and Firpo and Pinto (2016) ^43^, are not presented further.

**Gender difference effect of ICFCOA**

Although the above RIF-OLS regressions may, to some extent, reflect the differential performance between males and females in terms of their cognitive function inequality, they only provide a local approximation estimate. To better understand and confirm the existence of this inequality effect, we followed the estimation approach suggested by Firpo and Pinto (2016) ^43^ as follows.

We assume that the joint distribution function $F_{Y_{1},Y_{0},X,T}\left( \cdot\right)$ characterizes the relationship between the potential outcome variables $Y_{1}$ and $Y_{0}$, the exogenous explanatory variable vector $\mathbf{X}$, and the binary treatment variable $T$ (i.e., gender grouping). Although the potential outcome variables $Y_{1}$ and $Y_{0}$ could not be observed simultaneously, based on the unconfoundedness assumption and overlapping support proposed by Firpo and Pinto (2016) ^43^, we were able to define the distribution of observed outcomes across individuals of different gender groupings as:

$$\begin{aligned} F_{Y_{k}|T=k}=\int_{i\in k} F_{Y_{k}|X}dF_{X|T=k} for k=0,1\#\left( 10 \right) \end{aligned}$$

Furthermore, the fitted value $\hat{F}_{Y_{k}}$ of $F_{Y_{k}}$ can be estimated using the following equation based on the data obtained from reality.

$$\begin{aligned} \hat{F}_{Y_{k}}=\int_{i\in k} F_{Y_{k}|X}\omega_{k}\left( \mathbf{x} \right)dF_{X|T=k}\#\left( 11 \right) \end{aligned}$$

Where $\omega_{k}\left( \mathbf{x} \right)$ is the weighting factor, which can be obtained from the Bayesian formula combined with parameter estimation methods such as logit or probit models. After the weighting process described above, the overall treatment effect can be estimated using RIF regression.

$$\begin{aligned} \begin{aligned} \Delta\hat{v}=v\left( \hat{F}_{Y_{1}} \right)-v\left( \hat{F}_{Y_{0}} \right)=&\int_{i\in1} E\left[ RIF\left\{ y,v\left( \hat{F}_{Y_{1}} \right) \right\} | \boldsymbol{X}=\mathbf{x} \right]\hat{\omega}_{1}\left( \mathbf{x} \right)dF_{X|T=1}\left( \mathbf{x} \right)- \\ &\int_{i\in0} E\left[ RIF\left\{ y,v\left( \hat{F}_{Y_{0}} \right) \right\} | \boldsymbol{X}=\mathbf{x} \right]\hat{\omega}_{0}\left( \mathbf{x} \right)dF_{X|T=0}\left( \mathbf{x} \right) \end{aligned}\#\left( 12 \right) \end{aligned}$$

$$\begin{aligned} T\times RIF\left\{ y,v\left( \hat{F}_{Y_{1}} \right) \right\}+\left( 1-T \right)\times RIF\left\{ y,v\left( \hat{F}_{Y_{0}} \right) \right\}=b_{0}+b_{1}T+\varepsilon\#\left( 13 \right) \end{aligned}$$

Where $b_{1}$ is the difference in the distribution statistic due to gender grouping, i.e., the gender difference effect.

**Oaxaca-Blinder decomposition of ICFCOA**

While the above modified RIF regression, controlling for other factors, provides estimates of the gender effect of ICFCOA, an important issue is the need to analyze the source of this gender effect. To this end, this part of the study focuses on further decomposing the overall gender effects characterized by the distribution statistics of the measured inequality into an explainable part (i.e., the characteristic effect) and an unexplainable part (i.e., the coefficient effect) through RIF regressions^41^ using the Oaxaca-Blinder decomposition method. ^44 45^

It should be noted that a key step in applying the Oaxaca-Blinder decomposition method is the estimation of the counterfactual distribution statistic, $v_{c}$. According to Firpo et al. (2018) ^41^, $v_{c}$ is defined as follows:

$$\begin{aligned} v_{c}=v(F_{Y}^{c})=v(\int F_{Y|X,T=0}dF_{X|T=1})\#\left( 14 \right) \end{aligned}$$

Since the outcomes associated with the counterfactual distribution $F_{Y|X}^{c}$ and the distribution of the explanatory variable characteristics cannot be observed directly at the same time, it has to be approximated by the following equation:

$$\begin{aligned} F_{Y}^{c}=\int F_{Y|X,T=0}dF_{X|T=1}\cong\int F_{Y|X,T=0}dF_{X|T=0}\omega\left( \mathbf{X} \right)\#\left( 15 \right) \end{aligned}$$

The method of accessing the reweighting factor $\omega\left( \mathbf{X} \right)$ is similar to the method of estimating $\omega_{k}\left( \mathbf{x} \right)$ in equation (10). After obtaining the reweighting factor $\omega\left( \mathbf{X} \right)$, the identification of gender differences in the distribution statistics used to measure the degree of inequality and their decomposition can be achieved by using RIF regression and weighted least squares (WLS).

$$\begin{aligned} \begin{aligned} \Delta v&=\underset{\Delta v_{S}}{\underbrace{v_{1}-v_{c}}}+\underset{\Delta v_{X}}{\underbrace{v_{c}-v_{0}}} \\ &=E\left[ RIFy,v\left( F_{Y|T=1} \right) \right]-E\left[ RIFy,v\left( F_{Y}^{c} \right) \right]+E\left[ RIFy,v\left( F_{Y}^{c} \right) \right]-E\left[ RIFy,v\left( F_{Y|T=0} \right) \right] \\ &={\bar{\mathbf{X}}}^{1}\hat{\beta}^{1}-{\bar{\mathbf{X}}}^{c^{'}}\hat{\beta}^{c}+{\bar{\mathbf{X}}}^{c^{'}}\hat{\beta}^{c}-{\bar{\mathbf{X}}}^{0}\hat{\beta}^{0} \\ &=\underset{\Delta v_{s}^{p}}{\underbrace{{\bar{\mathbf{X}}}^{1^{'}}\left( \hat{\beta}^{1}-\hat{\beta}^{c} \right)}}+\underset{\Delta v_{s}^{e}}{\underbrace{\left( {\bar{\mathbf{X}}}^{1}-{\bar{\mathbf{X}}}^{c} \right)^{'}\hat{\beta}^{c}}}+\underset{\Delta v_{X}^{p}}{\underbrace{\left( {\bar{\mathbf{X}}}^{c}-{\bar{\mathbf{X}}}^{0} \right)^{'}\hat{\beta}^{0}}}+\underset{\Delta v_{X}^{e}}{\underbrace{{\bar{\mathbf{X}}}^{c^{'}}\left( \hat{\beta}^{c}-\hat{\beta}^{0} \right)}} \end{aligned}\#\left( 16 \right) \end{aligned}$$

Where $\Delta v_{S}$ is the difference caused by different rates of change in cognitive function, i.e., the coefficient effect, which is the unexplained part and can be subdivided into two parts: the pure coefficient effect $\Delta v_{s}^{p}$ and the reweighting error $\Delta v_{s}^{e}$. In contrast, $\Delta v_{X}$, which reflects differences due to individual characteristics of males and females, i.e., characteristic effect, belongs to the explainable part, which can be similarly subdivided into two parts: the pure characteristic effect $\Delta v_{X}^{p}$ and the model specification error $\Delta v_{X}^{e}$. The Oaxaca-Blinder decomposition based on the RIFs helps us to identify more clearly the possible sources of the gender differences that contribute to the ICFCOA.

**References**

1. Glisky EL. Brain Aging: Models, Methods, and Mechanisms. Boca Raton (FL): CRC Press/Taylor & Francis 2007.

2. Tymula A, Belmaker LAR, Ruderman L, et al. Like cognitive function, decision making across the life span shows profound age-related changes. *Proc Natl Acad Sci U S A* 2013;110:17143-48. doi: 10.1073/pnas.1309909110

3. Xu JF, Zhang YQ, Qiu CX, et al. Global and regional economic costs of dementia: a systematic review. *Lancet* 2017;390:S47-S47. doi: 10.1016/s0140-6736(17)33185-9

4. Meijer E, Casanova M, Kim H, et al. Economic costs of dementia in 11 countries in Europe: Estimates from nationally representative cohorts of a panel study. *Lancet Reg Health-Eur* 2022;20:12. doi: 10.1016/j.lanepe.2022.100445

5. Park DC, Polk TA, Mikels JA, et al. Cerebral aging: integration of brain and behavioral models of cognitive function. *Dialogues Clin Neurosci* 2022 doi: 10.31887/DCNS.2001.3.3/dcpark

6. Atalay K, Barrett GF, Staneya A. The effect of retirement on elderly cognitive functioning. *J Health Econ* 2019;66:37-53. doi: 10.1016/j.jhealeco.2019.04.006

7. Wang J, Xiao LD, Wang K, et al. Gender Differences in Cognitive Impairment among Rural Elderly in China. *Int J Environ Res Public Health* 2020;17:16. doi: 10.3390/ijerph17103724

8. Liang F, Fu JL, Moore JB, et al. Body Mass Index, Waist Circumference, and Cognitive Decline Among Chinese Older Adults: A Nationwide Retrospective Cohort Study. *Frontiers in Aging Neuroscience* 2022;14:9. doi: 10.3389/fnagi.2022.737532

9. Liu ZZ, Yang HQ, Chen SY, et al. The association between body mass index, waist circumference, waist-hip ratio and cognitive disorder in older adults. *J Public Health* 2019;41:305-12. doi: 10.1093/pubmed/fdy121

10. Wu SS, Lv XZ, Shen J, et al. Association between body mass index, its change and cognitive impairment among Chinese older adults: a community-based, 9-year prospective cohort study. *Eur J Epidemiol* 2021;36:1043-54. doi: 10.1007/s10654-021-00792-y

11. Xu PR, Wei R, Cheng BJ, et al. The association of marital status with cognitive function and the role of gender in Chinese community-dwelling older adults: a cross-sectional study. *Aging Clin Exp Res* 2021;33:2273-81. doi: 10.1007/s40520-020-01743-5

12. Shen S, Cheng JD, Li JP, et al. Association of marital status with cognitive function in Chinese hypertensive patients: a cross-sectional study. *BMC Psychiatry* 2022;22:11. doi: 10.1186/s12888-022-04159-9

13. Fernandez E, Schiaffino A, Borrell C, et al. Social class, education, and smoking cessation: Long-term follow-up of patients treated at a smoking cessation unit. *Nicotine Tob Res* 2006;8:29-36. doi: 10.1080/14622200500264432

14. Corley J, Gow AJ, Starr JM, et al. Smoking, childhood IQ and cognitive function in old age. *J Psychosom Res* 2012;73:132-38. doi: 10.1016/j.jpsychores.2012.03.006

15. Mons U, Schottker B, Muller H, et al. History of lifetime smoking, smoking cessation and cognitive function in the elderly population. *Eur J Epidemiol* 2013;28:823-31. doi: 10.1007/s10654-013-9840-9

16. Brennan SE, McDonald S, Page MJ, et al. Long-term effects of alcohol consumption on cognitive function: a systematic review and dose-response analysis of evidence published between 2007 and 2018. *Syst Rev* 2020;9:39. doi: 10.1186/s13643-019-1220-4

17. Banks J, Mazzonna F. The Effect of Education on Old Age Cognitive Abilities: Evidence from a Regression Discontinuity Design. *Econ J* 2012;122:418-48. doi: 10.1111/j.1468-0297.2012.02499.x

18. Park S, Choi B, Choi C, et al. Relationship between education, leisure activities, and cognitive functions in older adults. *Aging Ment Health* 2019;23:1651-60. doi: 10.1080/13607863.2018.1512083

19. Kuiper JS, Zuidersma M, Zuidema SU, et al. Social relationships and cognitive decline: a systematic review and meta-analysis of longitudinal cohort studies. *Int J Epidemiol* 2016;45:1169-206. doi: 10.1093/ije/dyw089

20. Elovainio M, Sommerlad A, Hakulinen C, et al. Structural social relations and cognitive ageing trajectories: evidence from the Whitehall II cohort study. *Int J Epidemiol* 2018;47:701-08. doi: 10.1093/ije/dyx209

21. Lautenschlager NT, Cox KL, Flicker L, et al. Effect of physical activity on cognitive function in older adults at risk for Alzheimer disease - A randomized trial. *JAMA* 2008;300:1027-37. doi: 10.1001/jama.300.9.1027

22. Karssemeijer EGA, Aaronson JA, Bossers WJ, et al. Positive effects of combined cognitive and physical exercise training on cognitive function in older adults with mild cognitive impairment or dementia: A meta-analysis. *Ageing Res Rev* 2017;40:75-83. doi: 10.1016/j.arr.2017.09.003

23. Duncan GJ, Magnuson K. Socioeconomic status and cognitive functioning: moving from correlation to causation. *Wiley Interdiscip Rev-Cogn Sci* 2012;3:377-86. doi: 10.1002/wcs.1176

24. Wu F, Guo YF, Zheng Y, et al. Social-Economic Status and Cognitive Performance among Chinese Aged 50 Years and Older. *PLoS ONE* 2016;11:9. doi: 10.1371/journal.pone.0166986

25. Huang GY, Xie Y, Xu HW. Cognitive Ability: Social Correlates and Consequences in Contemporary China. *Chin Sociol Rev* 2015;47:287-313. doi: 10.1080/21620555.2015.1032161

26. Yang L, Martikainen P, Silventoinen K, et al. Association of socioeconomic status and cognitive functioning change among elderly Chinese people. *Age Ageing* 2016;45:673-79. doi: 10.1093/ageing/afw107

27. Lv XZ, Li WY, Ma Y, et al. Cognitive decline and mortality among community-dwelling Chinese older people. *BMC Med* 2019;17:10. doi: 10.1186/s12916-019-1295-8

28. Yang F, Cao JL, Qian DF, et al. Stronger Increases in Cognitive Functions among Socio-Economically Disadvantaged Older Adults in China: A Longitudinal Analysis with Multiple Birth Cohorts. *Int J Environ Res Public Health* 2020;17:13. doi: 10.3390/ijerph17072418

29. Qi DW, Shi CH, Mao RY, et al. Sex-related associations between body height and cognitive impairment among low-income elderly adults in rural China: a population-based cross-sectional study. *Biol Sex Differ* 2021;12:8. doi: 10.1186/s13293-021-00408-w

30. Ahrenfeldt LJ, Lindahl-Jacobsen R, Rizzi S, et al. Comparison of cognitive and physical functioning of Europeans in 2004-05 and 2013. *Int J Epidemiol* 2018;47:1518-28. doi: 10.1093/ije/dyy094

31. Hayat SA, Luben R, Wareham N, et al. Cross-sectional and prospective relationship between occupational and leisure-time inactivity and cognitive function in an ageing population: the European Prospective Investigation into Cancer and Nutrition in Norfolk (EPIC-Norfolk) study. *Int J Epidemiol* 2020;49:1338-52. doi: 10.1093/ije/dyaa067

32. Olivera J, Andreoli F, Leist AK, et al. Inequality in old age cognition across the world. *Econ Hum Biol* 2018;29:179-88. doi: 10.1016/j.ehb.2018.03.002

33. Deng QW, Liu WB. Inequalities in cognitive impairment among older adults in China and the associated social determinants: a decomposition approach. *Int J Equity Health* 2021;20:14. doi: 10.1186/s12939-021-01422-5

34. Hou CB, Lin YN, Ren M, et al. Cognitive functioning transitions, health expectancies, and inequalities among elderly people in China: A nationwide longitudinal study. *Int J Geriatr Psychiatry* 2018;33:1635-44. doi: 10.1002/gps.4966

35. Lei XY, Hu YQ, McArdle JJ, et al. Gender Differences in Cognition among Older Adults in China. *J Hum Resour* 2012;47:951-71. doi: 10.3368/jhr.47.4.951

36. Xu HZ, Vorderstrasse AA, Dupre ME, et al. Gender differences in the association between migration and cognitive function among older adults in China and India. *Arch Gerontol Geriatr* 2019;81:31-38. doi: 10.1016/j.archger.2018.11.011

37. Koenker R, Bassett G. Regression Quantiles. *Econometrica* 1978;46:33-50. doi: 10.2307/1913643

38. Firpo S, Fortin NM, Lemieux T. Unconditional Quantile Regressions. *Econometrica* 2009;77:953-73. doi: 10.3982/ecta6822

39. Cowell FA, Flachalre E. Income distribution and inequality measurement: The problem of extreme values. *Journal of Econometrics* 2007;141:1044-72. doi: 10.1016/j.jeconom.2007.01.001

40. Rios-Avila F. Recentered influence functions (RIFs) in Stata: RIF regression and RIF decomposition. *Stata J* 2020;20:51-94. doi: 10.1177/1536867x20909690

41. Firpo SP, Fortin NM, Lemieux T. Decomposing Wage Distributions Using Recentered Influence Function Regressions. *Econometrics* 2018;6:40. doi: 10.3390/econometrics6020028

42. Choe C, Van Kerm P. Foreign Workers and the Wage Distribution: What Does the Influence Function Reveal? *Econometrics* 2018;6:26. doi: 10.3390/econometrics6030041

43. Firpo S, Pinto C. Identification and Estimation of Distributional Impacts of Interventions Using Changes in Inequality Measures. *J Appl Econom* 2016;31:457-86. doi: 10.1002/jae.2448

44. Oaxaca R. Male-female wage differentials in urban labor markets. *International economic review* 1973;14:693-709. doi: 10.2307/2525981

45. Blinder AS. Wage discrimination - reduced from and structural estimates. *J Hum Resour* 1973;8:436-55. doi: 10.2307/144855

**Table S1** RIF-UQR regression results of the factors influencing CFCOA

| Characteristics/Variables (Reference group) | Model 1 | | Model 2 | | Model 3 | | Model 4 | | Model 5 | | Model 6 | |
| --- | --- | --- | --- | --- | --- | --- | --- | --- | --- | --- | --- | --- |
|  | OLS | *P*-value | Q10 | *P*-value | Q25 | *P*-value | Q50 | *P*-value | Q75 | *P*-value | Q90 | *P*-value |
| Gender (Females) | 0.793 | ≤0.001 | 1.519 | ≤0.001 | 1.228 | ≤0.001 | 0.838 | ≤0.001 | 0.585 | ≤0.001 | 0.344 | 0.095 |
| Age | -0.097 | ≤0.001 | -0.134 | ≤0.001 | -0.122 | ≤0.001 | -0.101 | ≤0.001 | -0.078 | ≤0.001 | -0.067 | ≤0.001 |
| BMI (Normal: 18.5 ≤ BMI < 24) |  |  |  |  |  |  |  |  |  |  |  |  |
| Light (12 ≤ BMI < 18.5) | -0.434 | 0.027 | -0.491 | 0.322 | -0.765 | 0.046 | -0.566 | 0.057 | -0.367 | 0.125 | -0.351 | 0.127 |
| Heavy (24 ≤ BMI < 28) | 0.108 | 0.365 | 0.256 | 0.343 | 0.115 | 0.589 | 0.308 | 0.093 | 0.085 | 0.590 | -0.098 | 0.609 |
| Obese (28 ≤ BMI ≤ 60) | 0.244 | 0.262 | 0.168 | 0.696 | 0.322 | 0.392 | 0.608 | 0.069 | 0.360 | 0.197 | 0.159 | 0.648 |
| Waist | 0.007 | 0.320 | 0.023 | 0.172 | 0.007 | 0.621 | -0.007 | 0.556 | -0.006 | 0.515 | 0.022 | 0.056 |
| Subjective health (Poor) |  |  |  |  |  |  |  |  |  |  |  |  |
| Moderate | 0.280 | 0.005 | 0.071 | 0.782 | 0.121 | 0.521 | 0.475 | 0.003 | 0.314 | 0.012 | 0.430 | 0.002 |
| Excellent | 0.318 | 0.016 | -0.004 | 0.988 | 0.134 | 0.554 | 0.493 | 0.017 | 0.163 | 0.342 | 0.751 | ≤0.001 |
| Marital status (Not in marriage) | 0.190 | 0.127 | 0.149 | 0.616 | 0.630 | 0.005 | 0.328 | 0.079 | 0.010 | 0.950 | -0.117 | 0.508 |
| Smoking (Never) |  |  |  |  |  |  |  |  |  |  |  |  |
| Former | -0.373 | 0.005 | -0.887 | 0.006 | -0.589 | 0.014 | -0.242 | 0.256 | -0.217 | 0.252 | -0.204 | 0.376 |
| Long-term/Current | -0.475 | ≤0.001 | -0.646 | 0.020 | -0.633 | 0.007 | -0.614 | 0.002 | -0.313 | 0.067 | -0.267 | 0.170 |
| Drinking (Never) |  |  |  |  |  |  |  |  |  |  |  |  |
| Less than once a month | 0.013 | 0.928 | 0.221 | 0.493 | 0.420 | 0.096 | 0.076 | 0.735 | -0.298 | 0.140 | -0.390 | 0.103 |
| More than once a month | -0.003 | 0.981 | -0.052 | 0.827 | 0.026 | 0.900 | -0.050 | 0.788 | -0.035 | 0.822 | -0.069 | 0.703 |
| Education (No formal education) |  |  |  |  |  |  |  |  |  |  |  |  |
| Elementary school or below | 2.825 | ≤0.001 | 4.552 | ≤0.001 | 4.594 | ≤0.001 | 3.174 | ≤0.001 | 1.453 | ≤0.001 | 0.791 | ≤0.001 |
| Junior high school or above | 4.110 | ≤0.001 | 4.854 | ≤0.001 | 5.514 | ≤0.001 | 4.798 | ≤0.001 | 3.264 | ≤0.001 | 2.413 | ≤0.001 |
| Social interaction (Inactive) | 0.649 | ≤0.001 | 0.407 | 0.057 | 0.770 | ≤0.001 | 0.787 | ≤0.001 | 0.591 | ≤0.001 | 0.408 | 0.002 |
| Physical activity (Inactive) |  |  |  |  |  |  |  |  |  |  |  |  |
| Low to moderately active | 0.879 | ≤0.001 | 0.897 | 0.013 | 1.445 | ≤0.001 | 1.044 | ≤0.001 | 0.578 | ≤0.001 | 0.526 | 0.002 |
| Highly active | 0.817 | ≤0.001 | 0.526 | 0.226 | 1.435 | ≤0.001 | 1.070 | ≤0.001 | 0.431 | 0.036 | 0.589 | 0.006 |
| Family size (Small: 1 to 2) |  |  |  |  |  |  |  |  |  |  |  |  |
| Medium (3 to 4) | -0.076 | 0.460 | 0.183 | 0.422 | 0.093 | 0.613 | -0.221 | 0.137 | -0.070 | 0.599 | -0.064 | 0.679 |
| Large (5 or more) | -0.097 | 0.418 | 0.165 | 0.532 | -0.064 | 0.765 | -0.097 | 0.602 | -0.164 | 0.295 | -0.196 | 0.299 |
| Family socioeconomic status (Low) |  |  |  |  |  |  |  |  |  |  |  |  |
| Relatively low | 0.028 | 0.829 | 0.103 | 0.725 | 0.295 | 0.219 | 0.109 | 0.595 | -0.049 | 0.773 | -0.297 | 0.118 |
| Relatively high | -0.242 | 0.088 | -0.233 | 0.437 | -0.312 | 0.267 | -0.287 | 0.213 | -0.171 | 0.358 | -0.430 | 0.041 |
| High | -0.165 | 0.175 | -0.296 | 0.275 | -0.157 | 0.484 | -0.104 | 0.598 | -0.214 | 0.173 | -0.308 | 0.093 |
| Hukou (Agricultural hukou) | 0.540 | ≤0.001 | 0.134 | 0.631 | -0.082 | 0.746 | 0.783 | 0.008 | 0.991 | ≤0.001 | 0.963 | ≤0.001 |
| Constants | 12.122 | ≤0.001 | 7.536 | ≤0.001 | 9.681 | ≤0.001 | 13.244 | ≤0.001 | 16.284 | ≤0.001 | 15.508 | ≤0.001 |
| Work status FE | Yes | | Yes | | Yes | | Yes | | Yes | | Yes | |
| Community FE | Yes | | Yes | | Yes | | Yes | | Yes | | Yes | |
| Interviewed FE | Yes | | Yes | | Yes | | Yes | | Yes | | Yes | |
| Adjusted R-squared | 0.396 | | 0.159 | | 0.261 | | 0.279 | | 0.234 | | 0.143 | |
| RMSE | 2.673 | | 6.073 | | 4.855 | | 4.113 | | 3.522 | | 4.057 | |
| Average RIF | — | | 5.612 | | 8.345 | | 10.973 | | 13.258 | | 14.698 | |
| Observations | 4821 | | 4821 | | 4821 | | 4821 | | 4821 | | 4821 | |

CFCOA, cognitive function in Chinese older adults; BMI, body mass index; FE: fixed effect; RMSE, Root mean squared error; RIF, recentered influence function; UQR, unconditional quantile regression; OLS, ordinary least squares.

Standard errors and *P*-values for all models were obtained using bootstrap methods with 500 replications.

^*^*P* < 0.05, ^**^*P* < 0.01.

**Table S2** Results of gender-differentiated RIF-OLS regression of ICFCOA (Controls + IPW)

| Gender grouping | Model 1 | | Model 2 | | Model 3 | | Model 4 | | Model 5 | | Model 6 | | Model 7 | |
| --- | --- | --- | --- | --- | --- | --- | --- | --- | --- | --- | --- | --- | --- | --- |
|  | Mean | *P*-value | Q10 | *P*-value | Q90 | *P*-value | IQ-Range | *P*-value | IQ-Ratio | *P*-value | Gini | *P*-value | CV | *P*-value |
| Gender (Females) | 0.887 | ≤0.001 | 0.900 | 0.005 | 0.245 | 0.295 | -0.650 | 0.104 | -0.341 | 0.040 | -0.037 | ≤0.001 | -0.063 | ≤0.001 |
| Age | -0.136 | ≤0.001 | -0.134 | 0.002 | -0.098 | ≤0.001 | 0.034 | 0.573 | 0.046 | 0.186 | 0.004 | ≤0.001 | 0.007 | ≤0.001 |
| BMI (Normal: 18.5 ≤ BMI < 24) |  |  |  |  |  |  |  |  |  |  |  |  |  |  |
| Light (12 ≤ BMI < 18.5) | -0.453 | 0.265 | -0.486 | 0.567 | -0.375 | 0.376 | 0.103 | 0.916 | 0.145 | 0.765 | 0.021 | 0.366 | 0.035 | 0.371 |
| Heavy (24 ≤ BMI < 28) | 0.318 | 0.188 | -0.097 | 0.806 | 0.131 | 0.723 | 0.224 | 0.675 | 0.043 | 0.852 | -0.002 | 0.868 | -0.001 | 0.957 |
| Obese (28 ≤ BMI ≤ 60) | 0.135 | 0.705 | -0.133 | 0.803 | -0.296 | 0.554 | -0.167 | 0.817 | -0.008 | 0.978 | -0.009 | 0.586 | -0.012 | 0.649 |
| Waist | -0.003 | 0.807 | 0.016 | 0.467 | 0.028 | 0.299 | 0.012 | 0.744 | -0.001 | 0.936 | 0.001 | 0.377 | 0.001 | 0.411 |
| Subjective health (Poor) |  |  |  |  |  |  |  |  |  |  |  |  |  |  |
| Moderate | 0.605 | 0.026 | 0.274 | 0.515 | 0.143 | 0.697 | -0.123 | 0.839 | -0.071 | 0.799 | -0.017 | 0.139 | -0.027 | 0.160 |
| Excellent | 1.068 | ≤0.001 | 0.350 | 0.429 | 1.399 | 0.006 | 1.052 | 0.156 | 0.081 | 0.776 | -0.016 | 0.128 | -0.028 | 0.105 |
| Marital status (Not in marriage) | -0.155 | 0.616 | 0.699 | 0.271 | -0.859 | 0.064 | -1.548 | 0.044 | -0.487 | 0.224 | -0.021 | 0.180 | -0.033 | 0.227 |
| Smoking (Never) |  |  |  |  |  |  |  |  |  |  |  |  |  |  |
| Former | -0.301 | 0.179 | -0.746 | 0.153 | -0.798 | 0.012 | -0.055 | 0.929 | 0.265 | 0.526 | 0.007 | 0.586 | 0.014 | 0.521 |
| Long-term/Current | -0.714 | ≤0.001 | ≤0.001 | 1.000 | -0.424 | 0.225 | -0.432 | 0.373 | -0.146 | 0.461 | 0.021 | 0.073 | 0.036 | 0.080 |
| Drinking (Never) |  |  |  |  |  |  |  |  |  |  |  |  |  |  |
| Less than once a month | 0.262 | 0.325 | -0.059 | 0.867 | -0.633 | 0.070 | -0.580 | 0.244 | -0.137 | 0.476 | -0.024 | 0.023 | -0.036 | 0.029 |
| More than once a month | -0.409 | 0.170 | -0.258 | 0.666 | 0.209 | 0.655 | 0.468 | 0.548 | 0.211 | 0.658 | 0.023 | 0.114 | 0.043 | 0.083 |
| Education (No formal education) |  |  |  |  |  |  |  |  |  |  |  |  |  |  |
| Elementary school or below | 2.876 | ≤0.001 | 3.606 | ≤0.001 | 0.961 | ≤0.001 | -2.597 | ≤0.001 | -1.591 | ≤0.001 | -0.129 | ≤0.001 | -0.218 | ≤0.001 |
| Junior high school or above | 3.799 | ≤0.001 | 4.125 | ≤0.001 | 2.792 | ≤0.001 | -1.281 | 0.104 | -1.529 | 0.002 | -0.116 | ≤0.001 | -0.198 | ≤0.001 |
| Social interaction (Inactive) | 0.765 | ≤0.001 | 0.139 | 0.673 | 0.601 | 0.027 | 0.466 | 0.299 | 0.065 | 0.745 | -0.018 | 0.060 | -0.030 | 0.068 |
| Physical activity (Inactive) |  |  |  |  |  |  |  |  |  |  |  |  |  |  |
| Low to moderately active | 0.800 | ≤0.001 | 0.863 | 0.075 | 0.670 | 0.016 | -0.176 | 0.744 | -0.257 | 0.261 | -0.022 | 0.109 | -0.041 | 0.098 |
| Highly active | 0.728 | 0.011 | 0.575 | 0.298 | 0.677 | 0.082 | 0.117 | 0.854 | -0.106 | 0.715 | -0.016 | 0.382 | -0.025 | 0.443 |
| Family size (Small: 1 to 2) |  |  |  |  |  |  |  |  |  |  |  |  |  |  |
| Medium (3 to 4) | -0.358 | 0.166 | 0.106 | 0.786 | -0.035 | 0.918 | -0.137 | 0.804 | -0.044 | 0.867 | 0.016 | 0.181 | 0.024 | 0.243 |
| Large (5 or more) | -0.033 | 0.887 | -0.425 | 0.283 | 0.506 | 0.349 | 0.920 | 0.173 | 0.260 | 0.264 | 0.011 | 0.363 | 0.016 | 0.438 |
| Family socioeconomic status (Low) |  |  |  |  |  |  |  |  |  |  |  |  |  |  |
| Relatively low | -0.225 | 0.474 | -0.349 | 0.490 | -0.018 | 0.965 | 0.325 | 0.612 | 0.146 | 0.676 | 0.005 | 0.721 | 0.010 | 0.660 |
| Relatively high | -0.167 | 0.427 | -0.407 | 0.272 | -0.055 | 0.893 | 0.348 | 0.536 | 0.199 | 0.342 | 0.010 | 0.299 | 0.017 | 0.291 |
| High | -0.191 | 0.383 | -0.698 | 0.091 | 0.344 | 0.250 | 1.029 | 0.039 | 0.385 | 0.110 | 0.027 | 0.016 | 0.047 | 0.015 |
| Hukou (Agricultural hukou) | 1.218 | ≤0.001 | 0.332 | 0.210 | 1.670 | ≤0.001 | 1.343 | 0.020 | 0.149 | 0.468 | -0.011 | 0.179 | -0.018 | 0.179 |
| Constants | 15.587 | ≤0.001 | 9.175 | 0.010 | 16.785 | ≤0.001 | 7.643 | 0.148 | 1.154 | 0.680 | 0.019 | 0.818 | 0.053 | 0.703 |
| Adjusted R-squared | 0.374 | | 0.169 | | 0.142 | | 0.064 | | 0.105 | | 0.204 | | 0.197 | |
| RMSE | 2.770 | | 4.638 | | 4.550 | | 6.411 | | 2.455 | | 0.140 | | 0.241 | |
| Average RIF | 10.128 | | 5.714 | | 14.674 | | 8.963 | | 2.577 | | 0.195 | | 0.343 | |
| Observations | 4868 | | 4868 | | 4868 | | 4868 | | 4868 | | 4868 | | 4868 | |

ICFCOA, inequalities in cognitive function in Chinese older adults; IQ range, the 90-10th interquartile range; IQ ratio, the 90-10th interquartile ratio; Gini, the Gini coefficient; CV, the coefficient of variation; BMI, body mass index; RMSE, Root mean squared error; RIF, recentered influence function; OLS, ordinary least squares.

Standard errors and *P*-values for all models were obtained using bootstrap methods with 500 replications.

^*^*P* < 0.05, ^**^*P* < 0.01.

**Table S3** Results of gender-differentiated RIF-OLS regression of ICFCOA (Controls)

| Gender grouping | Model 1 | | Model 2 | | Model 3 | | Model 4 | | Model 5 | | Model 6 | | Model 7 | |
| --- | --- | --- | --- | --- | --- | --- | --- | --- | --- | --- | --- | --- | --- | --- |
|  | Mean | *P*-value | Q10 | *P*-value | Q90 | *P*-value | IQ-Range | *P*-value | IQ-Ratio | *P*-value | Gini | *P*-value | CV | *P*-value |
| Gender (Females) | 0.750 | ≤0.001 | 1.192 | ≤0.001 | 0.063 | 0.693 | -1.128 | ≤0.001 | -0.505 | ≤0.001 | -0.035 | ≤0.001 | -0.059 | ≤0.001 |
| Age | -0.105 | ≤0.001 | -0.153 | ≤0.001 | -0.072 | ≤0.001 | 0.081 | ≤0.001 | 0.063 | ≤0.001 | 0.004 | ≤0.001 | 0.007 | ≤0.001 |
| BMI (Normal: 18.5 ≤ BMI < 24) |  |  |  |  |  |  |  |  |  |  |  |  |  |  |
| Light (12 ≤ BMI < 18.5) | -0.348 | 0.066 | -0.618 | 0.229 | -0.341 | 0.105 | 0.275 | 0.621 | 0.092 | 0.686 | 0.008 | 0.470 | 0.015 | 0.462 |
| Heavy (24 ≤ BMI < 28) | 0.134 | 0.214 | -0.158 | 0.486 | -0.018 | 0.920 | 0.138 | 0.645 | -0.014 | 0.908 | -0.003 | 0.564 | -0.005 | 0.610 |
| Obese (28 ≤ BMI ≤ 60) | 0.166 | 0.392 | -0.406 | 0.287 | 0.378 | 0.272 | 0.782 | 0.133 | 0.225 | 0.289 | 0.010 | 0.364 | 0.019 | 0.289 |
| Waist | 0.012 | 0.069 | 0.025 | 0.095 | 0.011 | 0.266 | -0.013 | 0.475 | -0.007 | 0.338 | -0.001 | 0.114 | -0.001 | 0.090 |
| Subjective health (Poor) |  |  |  |  |  |  |  |  |  |  |  |  |  |  |
| Moderate | 0.391 | ≤0.001 | 0.524 | 0.026 | 0.491 | ≤0.001 | -0.031 | 0.910 | -0.105 | 0.383 | -0.007 | 0.246 | -0.011 | 0.272 |
| Excellent | 0.596 | ≤0.001 | 0.502 | 0.048 | 0.870 | ≤0.001 | 0.371 | 0.236 | -0.042 | 0.752 | -0.004 | 0.513 | -0.006 | 0.574 |
| Marital status (Not in marriage) | 0.217 | 0.050 | 0.315 | 0.227 | 0.042 | 0.812 | -0.273 | 0.378 | -0.075 | 0.572 | -0.007 | 0.234 | -0.010 | 0.310 |
| Smoking (Never) |  |  |  |  |  |  |  |  |  |  |  |  |  |  |
| Former | -0.376 | 0.002 | -0.490 | 0.100 | -0.318 | 0.109 | 0.171 | 0.642 | 0.169 | 0.233 | 0.013 | 0.049 | 0.021 | 0.052 |
| Long-term/Current | -0.531 | ≤0.001 | -0.616 | 0.011 | -0.334 | 0.058 | 0.279 | 0.345 | 0.173 | 0.114 | 0.021 | ≤0.001 | 0.036 | ≤0.001 |
| Drinking (Never) |  |  |  |  |  |  |  |  |  |  |  |  |  |  |
| Less than once a month | 0.038 | 0.762 | 0.209 | 0.494 | -0.202 | 0.370 | -0.410 | 0.277 | -0.118 | 0.396 | -0.014 | 0.043 | -0.022 | 0.051 |
| More than once a month | -0.063 | 0.531 | 0.228 | 0.326 | -0.194 | 0.204 | -0.422 | 0.130 | -0.119 | 0.215 | -0.007 | 0.208 | -0.010 | 0.261 |
| Education (No formal education) |  |  |  |  |  |  |  |  |  |  |  |  |  |  |
| Elementary school or below | 2.868 | ≤0.001 | 3.927 | ≤0.001 | 1.234 | ≤0.001 | -2.687 | ≤0.001 | -1.737 | ≤0.001 | -0.120 | ≤0.001 | -0.200 | ≤0.001 |
| Junior high school or above | 4.133 | ≤0.001 | 4.444 | ≤0.001 | 2.965 | ≤0.001 | -1.469 | ≤0.001 | -1.528 | ≤0.001 | -0.122 | ≤0.001 | -0.203 | ≤0.001 |
| Social interaction (Inactive) | 0.738 | ≤0.001 | 0.891 | ≤0.001 | 0.502 | ≤0.001 | -0.386 | 0.074 | -0.310 | ≤0.001 | -0.021 | ≤0.001 | -0.036 | ≤0.001 |
| Physical activity (Inactive) |  |  |  |  |  |  |  |  |  |  |  |  |  |  |
| Low to moderately active | 0.855 | ≤0.001 | 1.227 | ≤0.001 | 0.426 | 0.013 | -0.797 | 0.054 | -0.404 | 0.024 | -0.031 | ≤0.001 | -0.054 | ≤0.001 |
| Highly active | 0.628 | ≤0.001 | 0.901 | 0.027 | 0.251 | 0.198 | -0.647 | 0.143 | -0.277 | 0.152 | -0.022 | 0.028 | -0.036 | 0.037 |
| Family size (Small: 1 to 2) |  |  |  |  |  |  |  |  |  |  |  |  |  |  |
| Medium (3 to 4) | -0.230 | 0.011 | 0.096 | 0.619 | -0.283 | 0.047 | -0.379 | 0.099 | -0.114 | 0.243 | 0.004 | 0.393 | 0.005 | 0.503 |
| Large (5 or more) | -0.061 | 0.567 | 0.053 | 0.819 | -0.218 | 0.198 | -0.272 | 0.340 | -0.141 | 0.243 | -0.004 | 0.536 | -0.006 | 0.523 |
| Family socioeconomic status (Low) |  |  |  |  |  |  |  |  |  |  |  |  |  |  |
| Relatively low | 0.078 | 0.538 | 0.373 | 0.199 | -0.270 | 0.140 | -0.643 | 0.061 | -0.214 | 0.139 | -0.011 | 0.082 | -0.018 | 0.115 |
| Relatively high | -0.136 | 0.303 | 0.244 | 0.399 | -0.161 | 0.408 | -0.405 | 0.247 | -0.121 | 0.414 | 0.001 | 0.824 | 0.004 | 0.753 |
| High | 0.010 | 0.927 | -0.084 | 0.751 | 0.055 | 0.735 | 0.138 | 0.654 | 0.045 | 0.742 | 0.004 | 0.535 | 0.007 | 0.462 |
| Hukou (Agricultural hukou) | 0.944 | ≤0.001 | 0.431 | 0.025 | 1.393 | ≤0.001 | 0.965 | ≤0.001 | 0.000 | 0.997 | -0.005 | 0.314 | -0.008 | 0.327 |
| Constants | 11.956 | ≤0.001 | 8.255 | ≤0.001 | 16.198 | ≤0.001 | 7.945 | ≤0.001 | 1.152 | 0.242 | 0.124 | 0.004 | 0.221 | 0.004 |
| Adjusted R-squared | 0.360 | | 0.147 | | 0.126 | | 0.045 | | 0.092 | | 0.186 | | 0.177 | |
| RMSE | 2.758 | | 5.951 | | 4.169 | | 7.197 | | 3.037 | | 0.143 | | 0.246 | |
| Average RIF | 10.335 | | 5.834 | | 14.702 | | 8.869 | | 2.583 | | 0.186 | | 0.330 | |
| Observations | 4868 | | 4868 | | 4868 | | 4868 | | 4868 | | 4868 | | 4868 | |

ICFCOA, inequalities in cognitive function in Chinese older adults; IQ range, the 90-10th interquartile range; IQ ratio, the 90-10th interquartile ratio; Gini, the Gini coefficient; CV, the coefficient of variation; BMI, body mass index; RMSE, Root mean squared error; RIF, recentered influence function; OLS, ordinary least squares.

Standard errors and *P*-values for all models were obtained using bootstrap methods with 500 replications.

^*^*P* < 0.05, ^**^*P* < 0.01.

**Table S4** Results of gender-differentiated RIF-OLS regression of ICFCOA (No controls + IPW)

| Gender grouping | Model 1 | | Model 2 | | Model 3 | | Model 4 | | Model 5 | | Model 6 | | Model 7 | |
| --- | --- | --- | --- | --- | --- | --- | --- | --- | --- | --- | --- | --- | --- | --- |
|  | Mean | *P*-value | Q10 | *P*-value | Q90 | *P*-value | IQ-Range | *P*-value | IQ-Ratio | *P*-value | Gini | *P*-value | CV | *P*-value |
| Gender (Females) | 1.013 | ≤0.001 | 0.919 | 0.015 | 0.275 | 0.341 | -0.640 | 0.187 | -0.346 | 0.100 | -0.041 | ≤0.001 | -0.070 | ≤0.001 |
| Constants | 9.639 | ≤0.001 | 5.270 | ≤0.001 | 14.542 | ≤0.001 | 9.271 | ≤0.001 | 2.743 | ≤0.001 | 0.215 | ≤0.001 | 0.377 | ≤0.001 |
| Adjusted R-squared | 0.021 | | 0.008 | | 0.001 | | 0.002 | | 0.004 | | 0.017 | | 0.017 | |
| RMSE | 3.466 | | 5.067 | | 4.912 | | 6.621 | | 2.589 | | 0.155 | | 0.266 | |
| Average RIF | 10.128 | | 5.714 | | 14.674 | | 8.963 | | 2.577 | | 0.195 | | 0.343 | |
| Observations | 4868 | | 4868 | | 4868 | | 4868 | | 4868 | | 4868 | | 4868 | |

ICFCOA, inequalities in cognitive function in Chinese older adults; IQ range, the 90-10th interquartile range; IQ ratio, the 90-10th interquartile ratio; Gini, the Gini coefficient; CV, the coefficient of variation; BMI, body mass index; RMSE, Root mean squared error; RIF, recentered influence function; OLS, ordinary least squares.

Standard errors and *P*-values for all models were obtained using bootstrap methods with 500 replications.

^*^*P* < 0.05, ^**^*P* < 0.01.

**Table S5** Results of gender-differentiated RIF-OLS regression of ICFCOA (No controls)

| Gender grouping | Model 1 | | Model 2 | | Model 3 | | Model 4 | | Model 5 | | Model 6 | | Model 7 | |
| --- | --- | --- | --- | --- | --- | --- | --- | --- | --- | --- | --- | --- | --- | --- |
|  | Mean | *P*-value | Q10 | *P*-value | Q90 | *P*-value | IQ-Range | *P*-value | IQ-Ratio | *P*-value | Gini | *P*-value | CV | *P*-value |
| Gender (Females) | 1.278 | ≤0.001 | 1.893 | ≤0.001 | 0.326 | 0.009 | -1.566 | ≤0.001 | -0.797 | ≤0.001 | -0.054 | ≤0.001 | -0.088 | ≤0.001 |
| Constants | 9.651 | ≤0.001 | 4.821 | ≤0.001 | 14.527 | ≤0.001 | 9.706 | ≤0.001 | 3.009 | ≤0.001 | 0.215 | ≤0.001 | 0.377 | ≤0.001 |
| Adjusted R-squared | 0.034 | | 0.021 | | 0.001 | | 0.011 | | 0.015 | | 0.028 | | 0.026 | |
| RMSE | 3.389 | | 6.374 | | 4.456 | | 7.324 | | 3.162 | | 0.156 | | 0.267 | |
| Average RIF | 10.335 | | 5.834 | | 14.702 | | 8.869 | | 2.583 | | 0.186 | | 0.330 | |
| Observations | 4868 | | 4868 | | 4868 | | 4868 | | 4868 | | 4868 | | 4868 | |

ICFCOA, inequalities in cognitive function in Chinese older adults; IQ range, the 90-10th interquartile range; IQ ratio, the 90-10th interquartile ratio; Gini, the Gini coefficient; CV, the coefficient of variation; BMI, body mass index; RMSE, Root mean squared error; RIF, recentered influence function; OLS, ordinary least squares.

Standard errors and *P*-values for all models were obtained using bootstrap methods with 500 replications.

^*^*P* < 0.05, ^**^*P* < 0.01.

**Table S6** Results of gender-differentiated PSM regression of ICFCOA

| Gender grouping | Model 1^a^:  Nearest neighbor | | Model 2^b^:  Radius | | Model 3^c^:  Kernel | | Model 4^d^: Local  linear regression | |
| --- | --- | --- | --- | --- | --- | --- | --- | --- |
|  | ATT 1 | T-Stat | ATT 2 | T-Stat | ATT 3 | T-Stat | ATT 4 | T-Stat |
| Mean | 0.930^*^ | 2.33 | 0.990^*^ | 2.57 | 0.888^**^ | 3.01 | 1.064^*^ | 2.49 |
| 10th quantile | 1.688^**^ | 2.79 | 1.786^**^ | 2.93 | 1.547^**^ | 3.29 | 1.741^**^ | 2.89 |
| 90th quantile | 0.139 | 0.24 | -0.007 | -0.01 | -0.173 | -0.42 | -0.126 | -0.19 |
| 90-10th IQ range | -1.547^*^ | -1.97 | -1.790^*^ | -2.36 | -1.718^**^ | -2.94 | -1.865^*^ | -2.23 |
| 90-10th IQ ratio | -0.697 | -1.72 | -0.795 | -1.95 | -0.670^*^ | -2.16 | -0.791 | -1.93 |
| Gini | -0.048^**^ | -2.70 | -0.054^**^ | -3.04 | -0.046^**^ | -3.41 | -0.055^**^ | -3.00 |
| CV | -0.083^**^ | -2.73 | -0.092^**^ | -3.04 | -0.080^**^ | -3.44 | -0.094^**^ | -3.02 |
| Pseudo R-squared | 0.485 | | 0.485 | | 0.485 | | 0.485 | |
| Observations | 4868 | | 4868 | | 4868 | | 4868 | |

ICFCOA, inequalities in cognitive function in Chinese older adults; IQ range, the 90-10th interquartile range; IQ ratio, the 90-10th interquartile ratio; Gini, the Gini coefficient; CV, the coefficient of variation; PSM, propensity score matching; ATT, average treatment effect.

^a^ In Model 1, using nearest neighbor matching method.

^b^ In Model 2, using radius matching method.

^c^ In Model 3, using kernel matching method.

^d^ In Model 4, using local linear regression matching method.

^*^*P* < 0.05, ^**^*P* < 0.01.

**Table S7** Results of Oaxaca-Blinder decomposition of gender differences in ICFCOA

| Characteristics/Variables  (Reference group) | Model 1 | | Model 2 | | | |
| --- | --- | --- | --- | --- | --- | --- |
|  | IQ Range | *P*-value | IQ Ratio | | *P*-value | |
| Overall |  |  |  | |  | |
| Female group | 9.706 | ≤0.001 | 3.009 | | ≤0.001 | |
| Counterfactual group | 8.709 | ≤0.001 | 2.437 | | ≤0.001 | |
| Male group | 8.140 | ≤0.001 | 2.212 | | ≤0.001 | |
| Female group | 1.566 | ≤0.001 | 0.797 | | ≤0.001 | |
| Total characteristic effect | 0.569 | 0.064 | 0.225 | | 0.017 | |
| Total coefficient effect | 0.997 | 0.003 | 0.572 | | ≤0.001 | |
| Explainable part |  |  |  | |  | |
| Total characteristic effect | 0.569 | 0.064 | 0.225 | | 0.017 | |
| Pure characteristic effect | 1.065 | 0.003 | 0.372 | | 0.002 | |
| Model specification error | -0.496 | 0.163 | -0.147 | | 0.188 | |
| Pure characteristic effect |  |  |  | |  | |
| Age | -0.038 | 0.202 | -0.021 | | 0.144 | |
| BMI (Normal: 18.5 ≤ BMI < 24) |  |  |  | |  | |
| Light (12 ≤ BMI < 18.5) | -0.006 | 0.749 | -0.002 | | 0.729 | |
| Heavy (24 ≤ BMI < 28) | 0.025 | 0.482 | 0.013 | | 0.268 | |
| Obese (28 ≤ BMI ≤ 60) | 0.005 | 0.769 | 0.002 | | 0.752 | |
| Waist | 0.004 | 0.913 | -0.008 | | 0.512 | |
| Subjective health (Poor) |  |  |  | |  | |
| Moderate | 0.005 | 0.746 | 0.003 | | 0.675 | |
| Excellent | -0.006 | 0.773 | 0.005 | | 0.438 | |
| Marital status (Not in marriage) | 0.178 | 0.031 | 0.050 | | 0.057 | |
| Smoking (Never) |  |  |  | |  | |
| Former | -0.112 | 0.222 | -0.046 | | 0.092 | |
| Long-term/Current | -0.142 | 0.329 | -0.082 | | 0.062 | |
| Drinking (Never) |  |  |  | |  | |
| Less than once a month | 0.005 | 0.793 | 0.000 | | 0.934 | |
| More than once a month | 0.062 | 0.539 | 0.007 | | 0.822 | |
| Education (No formal education) |  |  |  | |  | |
| Elementary school or below | 0.428 | 0.004 | 0.156 | | 0.004 | |
| Junior high school or above | 0.728 | ≤0.001 | 0.304 | | ≤0.001 | |
| Social interaction (Inactive) | 0.014 | 0.443 | 0.008 | | 0.389 | |
| Physical activity (Inactive) |  |  |  | |  | |
| Low to moderately active | -0.101 | 0.082 | -0.042 | | 0.048 | |
| Highly active | 0.069 | 0.199 | 0.033 | | 0.080 | |
| Family size (Small: 1 to 2) |  |  |  | |  | |
| Medium (3 to 4) | 0.004 | 0.749 | 0.001 | | 0.815 | |
| Large (5 or more) | -0.007 | 0.611 | -0.002 | | 0.596 | |
| Family socioeconomic status (Low) |  |  |  | |  | |
| Relatively low | -0.007 | 0.676 | -0.002 | | 0.720 | |
| Relatively high | 0.003 | 0.834 | 0.001 | | 0.868 | |
| High | 0.001 | 0.915 | 0.001 | | 0.809 | |
| Hukou (Agricultural hukou) | -0.051 | 0.060 | -0.005 | | 0.440 | |
| Model specification error |  |  |  | |  | |
| Age | 0.195 | 0.960 | -0.242 | | 0.856 | |
| BMI (Normal: 18.5 ≤ BMI < 24) |  |  |  | |  | |
| Light (12 ≤ BMI < 18.5) | -0.029 | 0.753 | -0.024 | | 0.466 | |
| Heavy (24 ≤ BMI < 28) | 0.223 | 0.495 | 0.024 | | 0.814 | |
| Obese (28 ≤ BMI ≤ 60) | 0.014 | 0.889 | 0.019 | | 0.563 | |
| Waist | 4.203 | 0.392 | 0.885 | | 0.542 | |
| Subjective health (Poor) |  |  |  | |  | |
| Moderate | -0.080 | 0.849 | -0.004 | | 0.975 | |
| Excellent | -0.039 | 0.826 | -0.002 | | 0.970 | |
| Marital status (Not in marriage) | -0.176 | 0.801 | 0.087 | | 0.669 | |
| Smoking (Never) |  |  |  | |  | |
| Former | 0.012 | 0.662 | 0.008 | | 0.449 | |
| Long-term/Current | 0.003 | 0.934 | 0.007 | | 0.584 | |
| Drinking (Never) |  |  |  | |  | |
| Less than once a month | -0.036 | 0.635 | -0.007 | | 0.809 | |
| More than once a month | 0.064 | 0.385 | 0.028 | | 0.338 | |
| Education (No formal education) |  |  |  | |  | |
| Elementary school or below | 1.999 | ≤0.001 | 0.579 | | 0.001 | |
| Junior high school or above | 0.837 | ≤0.001 | 0.228 | | ≤0.001 | |
| Social interaction (Inactive) | 0.721 | 0.040 | 0.312 | | 0.010 | |
| Physical activity (Inactive) |  |  |  | |  | |
| Low to moderately active | 1.199 | 0.106 | 0.200 | | 0.475 | |
| Highly active | 0.374 | 0.031 | 0.079 | | 0.222 | |
| Family size (Small: 1 to 2) |  |  |  | |  | |
| Medium (3 to 4) | 0.327 | 0.148 | 0.091 | | 0.185 | |
| Large (5 or more) | 0.056 | 0.725 | 0.054 | | 0.363 | |
| Family socioeconomic status (Low) |  |  |  | |  | |
| Relatively low | 0.496 | 0.020 | 0.112 | | 0.082 | |
| Relatively high | 0.126 | 0.435 | 0.021 | | 0.708 | |
| High | -0.120 | 0.750 | -0.075 | | 0.567 | |
| Hukou (Agricultural hukou) | -0.005 | 0.968 | 0.008 | | 0.840 | |
| Constants | -10.859 | 0.035 | -2.536 | | 0.105 | |
| Unexplainable part |  |  |  | |  | |
| Total coefficient effect | 0.997 | 0.003 | 0.572 | | ≤0.001 | |
| Pure coefficient effect | 0.015 | 0.906 | 0.017 | | 0.683 | |
| Reweighting error | 0.982 | 0.004 | 0.555 | | ≤0.001 | |
| Pure coefficient effect |  |  |  | |  | |
| Age | 2.164 | 0.611 | 4.579 | | 0.015 | |
| BMI (Normal: 18.5 ≤ BMI < 24) |  |  |  | |  | |
| Light (12 ≤ BMI < 18.5) | -0.041 | 0.569 | -0.001 | | 0.973 | |
| Heavy (24 ≤ BMI < 28) | -0.389 | 0.303 | -0.146 | | 0.264 | |
| Obese (28 ≤ BMI ≤ 60) | 0.036 | 0.793 | -0.004 | | 0.938 | |
| Waist | -6.362 | 0.219 | -1.389 | | 0.434 | |
| Subjective health (Poor) |  |  |  | |  | |
| Moderate | 0.466 | 0.335 | 0.118 | | 0.502 | |
| Excellent | 0.114 | 0.527 | 0.029 | | 0.674 | |
| Marital status (Not in marriage) | 1.676 | 0.032 | 0.370 | | 0.159 | |
| Smoking (Never) |  |  |  | |  | |
| Former | -0.041 | 0.339 | -0.008 | | 0.709 | |
| Long-term/Current | -0.028 | 0.549 | -0.021 | | 0.300 | |
| Drinking (Never) |  |  |  | |  | |
| Less than once a month | 0.014 | 0.825 | -0.014 | | 0.591 | |
| More than once a month | -0.077 | 0.278 | -0.052 | | 0.087 | |
| Education (No formal education) |  |  |  | |  | |
| Elementary school or below | -0.330 | 0.454 | -0.496 | | 0.007 | |
| Junior high school or above | 0.004 | 0.984 | -0.107 | | 0.111 | |
| Social interaction (Inactive) | -0.629 | 0.119 | -0.339 | | 0.030 | |
| Physical activity (Inactive) |  |  |  | |  | |
| Low to moderately active | -0.142 | 0.855 | 0.107 | | 0.749 | |
| Highly active | -0.284 | 0.193 | -0.024 | | 0.793 | |
| Family size (Small: 1 to 2) |  |  |  | |  | |
| Medium (3 to 4) | -0.445 | 0.079 | -0.128 | | 0.144 | |
| Large (5 or more) | -0.293 | 0.121 | -0.158 | | 0.038 | |
| Family socioeconomic status (Low) |  |  |  | |  | |
| Relatively low | -0.466 | 0.017 | -0.139 | | 0.065 | |
| Relatively high | -0.033 | 0.844 | -0.026 | | 0.708 | |
| High | 0.154 | 0.711 | 0.026 | | 0.876 | |
| Hukou (Agricultural hukou) | -0.038 | 0.810 | -0.058 | | 0.293 | |
| Constants | 5.952 | 0.289 | -1.562 | | 0.462 | |
| Reweighting error |  |  |  | |  | |
| Age | -0.021 | 0.459 | -0.010 | | 0.391 | |
| BMI (Normal: 18.5 ≤ BMI < 24) |  |  |  | |  | |
| Light (12 ≤ BMI < 18.5) | -0.003 | 0.931 | 0.002 | | 0.832 | |
| Heavy (24 ≤ BMI < 28) | 0.009 | 0.790 | 0.002 | | 0.826 | |
| Obese (28 ≤ BMI ≤ 60) | 0.008 | 0.767 | 0.006 | | 0.514 | |
| Waist | 0.003 | 0.913 | 0.000 | | 0.963 | |
| Subjective health (Poor) |  |  | |  | |  |
| Moderate | -0.007 | 0.783 | | -0.003 | | 0.736 |
| Excellent | 0.001 | 0.973 | | 0.001 | | 0.851 |
| Marital status (Not in marriage) | -0.041 | 0.434 | | -0.007 | | 0.559 |
| Smoking (Never) |  |  | |  | |  |
| Former | -0.002 | 0.553 | | -0.001 | | 0.485 |
| Long-term/Current | -0.002 | 0.586 | | -0.001 | | 0.336 |
| Drinking (Never) |  |  | |  | |  |
| Less than once a month | 0.011 | 0.614 | | 0.001 | | 0.854 |
| More than once a month | -0.006 | 0.678 | | -0.003 | | 0.620 |
| Education (No formal education) |  |  | |  | |  |
| Elementary school or below | 0.014 | 0.723 | | 0.011 | | 0.605 |
| Junior high school or above | 0.008 | 0.661 | | -0.001 | | 0.765 |
| Social interaction (Inactive) | 0.035 | 0.366 | | 0.013 | | 0.336 |
| Physical activity (Inactive) |  |  | |  | |  |
| Low to moderately active | -0.003 | 0.908 | | 0.006 | | 0.600 |
| Highly active | 0.018 | 0.548 | | 0.001 | | 0.898 |
| Family size (Small: 1 to 2) |  |  | |  | |  |
| Medium (3 to 4) | 0.012 | 0.605 | | 0.003 | | 0.595 |
| Large (5 or more) | 0.011 | 0.637 | | 0.007 | | 0.489 |
| Family socioeconomic status (Low) |  |  | |  | |  |
| Relatively low | -0.048 | 0.308 | | -0.011 | | 0.396 |
| Relatively high | 0.000 | 0.993 | | 0.000 | | 0.999 |
| High | -0.004 | 0.890 | | -0.002 | | 0.824 |
| Hukou (Agricultural hukou) | 0.020 | 0.393 | | 0.003 | | 0.616 |
| Observations | 4868 | | | 4868 | | |

ICFCOA, inequalities in cognitive function in Chinese older adults; IQ range, the 90-10th interquartile range; IQ ratio, the 90-10th interquartile ratio.

Standard errors and *P*-values for all models were obtained using bootstrap methods with 500 replications.

^*^*P* < 0.05, ^**^*P* < 0.01.
